# A 3D-Printable Dataset for Fair Testing and Comparisons of Tactile Sensors

**DOI:** 10.3390/s26206590

**Published:** 2026-10-17

**Authors:** Dexter R. Shepherd, Nicolas Herzig, Phil Husbands, Chris Johnson, Oluwaseyi Oladipupo Jesusanmi, William Kimbell, Andrew Philippides

**Affiliations:** 1 AI Group, Department of Informatics, University of Sussex, Brighton BN1 9RH, UK; p.husbands@sussex.ac.uk (P.H.); o.jesusanmi@sussex.ac.uk (O.O.J.);; 2 Department of Engineering, University of Sussex, Brighton BN1 9RH, UK; 3 Sussex Neuroscience, School of Life Sciences, University of Sussex, Brighton BN1 9RH, UK

**Keywords:** tactile sensors, 3D printing, dataset, haptics, benchmark, robotics

## Abstract

Existing texture datasets for tactile sensing primarily consist of sensor readings from a specific sensor interacting with available surfaces/objects rather than describing the textures themselves, limiting fair comparison between tactile sensors and hindering reproducible research. In this work, we introduce a six-piece digital dataset of mathematically defined textures designed to be fabricated reliably across different research groups. The dataset consists of surface patterns derived from combinations of sine-waves, giving controlled variation in spatial frequency, amplitude, and directional structure. To evaluate the physical repeatability of this framework, we present a validation methodology applied to the six-textures. We evaluate the reproducibility of these textures across three popular 3D printers and filament types in multiple ways. We first use an RGBD D405 stereoscopic camera to scan the 3D structure to assess physical differences between repeat prints. To see how physical differences affect a typical tactile sensor, we next calculate the difference in images captured using an optical TacTip sensor under controlled contact conditions. Our results show that print quality, particularly peak sharpness and stringing, affects tactile variance, with the Bambu P1P/PLA- and Form 3 resin configurations producing more consistent prints than the Ender-3/PLA+ configuration. To assess potential downstream effects of the variance, we train two types of classifier, random forest classifiers and neural networks, both with PCA preprocessing and data augmentation, to classify textures. By training on one batch of textures, but testing on different unseen batches, we further assessed how similar different batches of prints are. These experiments demonstrate that the Bambu P1P/PLA- and Form 3 resin configurations support both strong within-batch classification and cross-batch generalisation, reflecting the fact that these configurations had low physical and tactile image variance. This work establishes the first openly available, six-texture digital library paired with a metrology pipeline designed to be sensor-independent, providing an open foundation that enables researchers to replicate textures and fairly compare tactile sensing technologies, while our pipeline has been tested on only one tactile sensor (TacTip) and two classifier types, we hope future research will compare many other combinations of sensing technologies.

## 1. Introduction

While texture classification is an important topic in robotics [1,2,3,4,5,6,7] and is used as a benchmark task [8], fairly comparing texture classification performance between different tactile sensors is difficult due to an inability to reliably reproduce the textures themselves. Existing approaches typically rely on self-collected datasets from available objects [9,10], which limits the accuracy and fairness of comparisons with newly developed sensors. Indeed, most available texture datasets consist of sensor readings from specific tactile sensors [11,12,13], rather than the textures themselves. This limits their use to classifier optimisation or transfer learning [14], rather than allowing a direct comparison with other sensors. This paper fills this gap by designing a set of textures that can be 3D-printed by researchers, and supports its physical reproducibility by assessing the variability introduced by different printers and filament types.

Standardised datasets have been used in other fields. For example, 3D-printed textures have been employed by Tymms et al. to measure human tactile perception [15]. However, this task is aimed at investigating the resolution of human sensing and consists of similar geometric patterns. Although it could make a good benchmark for determining the minimal resolution of tactile sensors, it does not evaluate different textures; so we require something different for texture classification. In object classification, 3D datasets of objects exist [16], which allow the objects to be imported into simulations [17,18]. However, little work has been done with physical instantiations aside from random objects [8,19], and Braille [20,21,22]. The latter is a good example of a standardised and reproducible dataset, which is the goal of this paper.

Following on from the ideas presented in [15,20,21], we propose a 3D-printed dataset of textures that can be used to compare the performance of various tactile sensors. To do this, we use equation-generated texture blocks that can be 3D printed. We then use a TacTip to gather a visual dataset across different printer–filament combinations to determine print variance. Importantly, 3D-printing quality varies between different printers and filaments, as well as being affected by other factors that are not always easy to control, such as humidity [23]. We therefore investigate how reproducible a dataset is across popular printers and a range of filament types. We investigate this variation using three methods: firstly, by scanning repeats of the printed textures using a depth camera and analysing differences; secondly, by comparing the differences between TacTip images from repeat prints; thirdly, we train texture classifiers on one repeat but assess accuracy on the unseen repeats, to assess the impact of dataset variation on classification. In so doing, this work paves the way for fair and reproducible tactile sensor comparisons.

## 2. Methods

### 2.1. Creation of Textures

We considered various approaches for generating textures in this dataset. Initially, we explored 3D-scanning real-world surfaces to better reflect natural textures. However, this introduced variability, including non-uniformity within a single sample. As a result, different regions of the same texture could produce inconsistent sensor responses depending on the contact location, reducing reproducibility and making controlled comparisons difficult.

To address this, we instead draw inspiration from the USAF calibration test [24], which employs well-defined geometric patterns that can be systematically scaled without loss of fidelity. By using mathematically generated patterns, we ensure that each texture is uniform and precisely controllable. This allows consistent sampling across the surface, improves reproducibility, and enables more rigorous benchmarking of sensor performance across well-defined stimulus conditions.

The six textures, the number kept small for practicality, were deliberately designed to share the same fundamental spatial period while differing in their higher-order spectral content and surface topology (e.g., ridged, rounded, square-profile, and point-like asperity structures). This design creates textures that are similar enough to present a meaningful discrimination challenge, while still spanning representative classes of surface geometries encountered in tactile sensing. We also intentionally kept the number of textures small to provide a simple, scalable benchmark that is practical to reproduce and extend. This philosophy mirrors established resolution standards in other sensing domains, such as ISO 12233 [25] and the 1951 USAF resolution target [24] favouring simplicity, reproducibility, and diagnostic value rather than exhaustive coverage of all possible stimuli.

Following on from the ideas presented in [15,20,21], we propose a two-phase benchmarking framework to enable the standardised comparison of various tactile sensors. First, we introduce an open-source digital library of six equation-generated texture blocks spanning a compact, deliberately challenging initial benchmark set with a graded range of structural profiles. Second, we establish a validation methodology to quantify and minimise physical fabrication variance, evaluating six textures across popular 3D printers and filament types. Because 3D-printing quality is inherently susceptible to machine/filament-specific errors and environmental variables like humidity [23], we investigate physical dataset reproducibility using complementary methods: 3D optical scanning via a depth camera to quantify the geometric repeatability of repeat prints of the same texture; comparing the differences between tactile images gathered with a TacTip sensor from repeat prints of the textures; training machine learning classifiers on one repeat of the visual tactile datasets gathered, but testing them on the other four unseen repeats. In doing so, this work provides both a compact, deliberately challenging benchmark set and an empirical roadmap for achieving consistent physical replication.

The equations used to generate the textures are given below, representing a wide range of scalable surfaces. Some textures are deliberately similar to make discrimination between them challenging, a desirable property of a test suite for benchmarking sensors and classification techniques.

**Textures One and Two**

Textures One and Two are generated using Equation (1). The parameters $A_{1}$ and $A_{2}$ represent the amplitudes of sine waves in the *x*- and *y*-directions, respectively, $f_{1}$ and $f_{2}$ denote the frequencies of the sine components along the *x*- and *y*-axes and ϕ is a phase offset applied to the sine term involving *y*. The variables *x* and *y* are spatial coordinate inputs, and the output $z_{1}$, the sum of two sine functions modulated by their respective amplitudes, frequencies, and phase, gives the height of the surface.

(1) $$z_{1}=A_{1}\sin\left(f_{1}x\right)+A_{2}\sin\left(f_{2}y+\phi \right)$$

**Figure 1:**
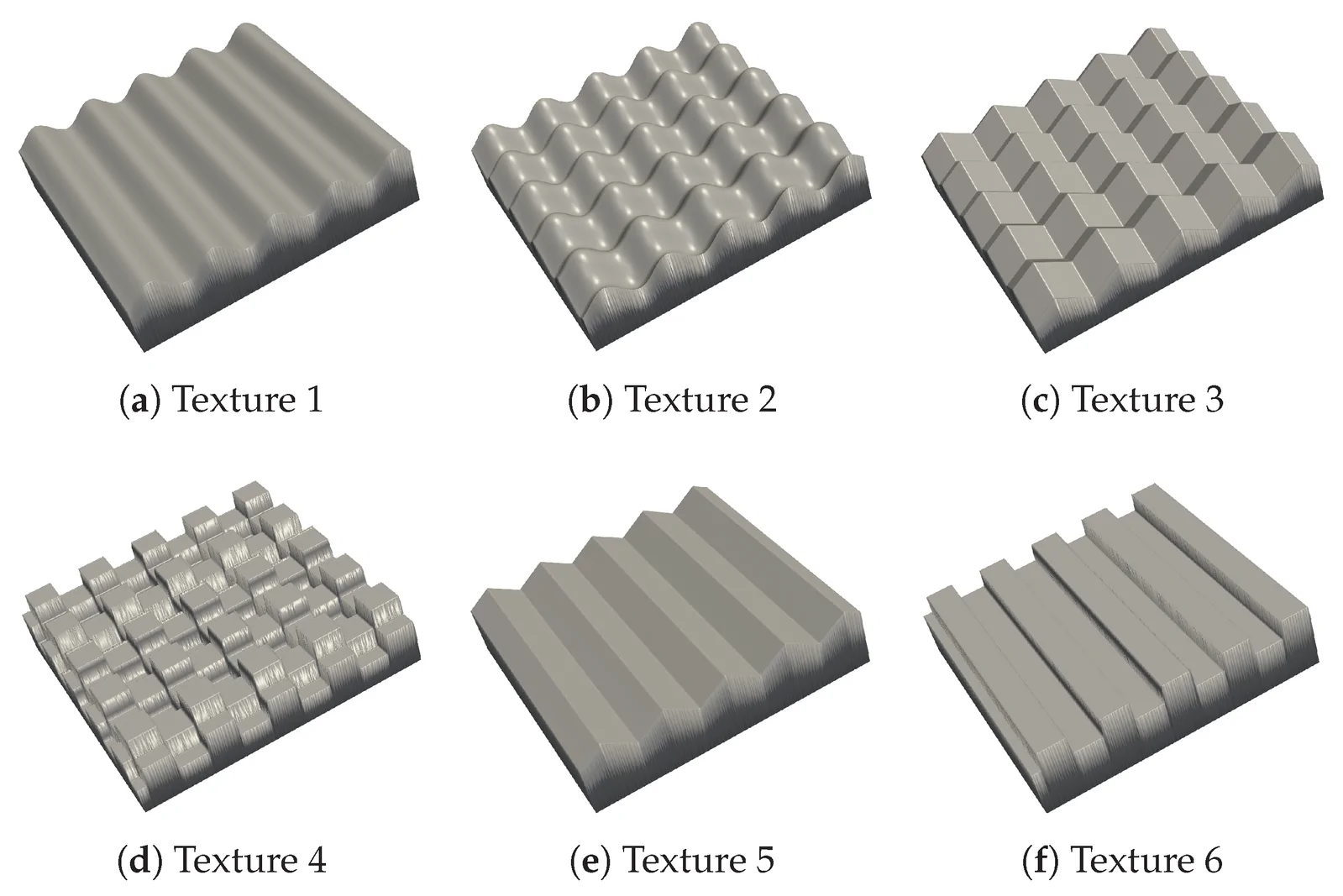
The 3D-printable textures generated from the equations listed above.

For Texture One, the parameter values are $A_{1}=1$, $A_{2}=0$, $f_{1}=1$, $f_{2}=1$, ϕ=π/2 with x∈[0,6π+0.5] and y∈[0,6π+1.2], producing sinusoidal variations in one direction only. The resulting surface is shown in Figure 1.

For Texture Two, the parameter values are $A_{1}=1$, $A_{2}=1$, $f_{1}=1$, $f_{2}=1$, ϕ=π/2 with x∈[0,6π+0.5] and y∈[0,6π+1.2], introducing sinusoidal variation in both dimensions.

**Texture Three**

For Texture Three, we use the function $z_{3}$ defined over a grid using the following summation (Equation (2)):

(2) $$z_{3}=\sum_{\begin{array}{c} i=1\\ i\;\mathrm{odd} \end{array}}^{N}\left(A_{i}\cdot \sin\left(ix\right)+A_{i}\cdot \sin\left(iy\right)\right)$$

In this expression, *N* is the number of terms in the summation, taken over odd integers from 1 to *N*. For our texture, we used N=25. Each term in the series uses the coefficient $A_{i}$, which controls the amplitude of the sine wave and is defined by Equation (3).

(3) $$A_{i}=\frac{8}{\pi^{2}}\cdot \frac{{\left(-1\right)}^{\frac{i-1}{2}}}{i^{2}}$$

The sine functions sin(ix) and sin(iy) vary with spatial coordinates *x* and *y*, respectively, and share the same frequency f=i and amplitude $A_{i}$. The ranges of *x* and *y* are the same as for Textures One and Two. These ranges are used for all the textures described in this section.

As $z_{3}$ is built-up by adding sine wave components in both the *x*- and *y*-directions, the result is a 2D symmetrical pattern (see Figure 1).

**Texture Four**

Texture Four uses the same equation as Texture Three (Equation (2)), but with a modified amplitude coefficient, $A_{i}$, given by Equation (4).

(4) $$A_{i}=\frac{4}{\pi i}$$

The decay and lack of sign alternation lead to sharper features, with more pronounced ridges and edges (see Figure 1).

**Texture Five**

Texture Five is generated by the function $z_{5}$, shown in Equation (5).

(5) $$z_{5}=\sum_{\begin{array}{c} i=1\\ i\;\mathrm{odd} \end{array}}^{N}\left(A_{i}\cdot \sin\left(ix\right)\right)$$

where $A_{i}$ is defined by Equation (3) (as for Texture Three). With variation along one axis only, the shape has an extruded profile (see Figure 1).

**Texture Six**

Texture six uses the same function as Texture Five (Equation (5)), but with the coefficient, $A_{i}$, now defined by Equation (4), as in Texture Four.

### 2.2. Conversion to STL

**Figure 2:**
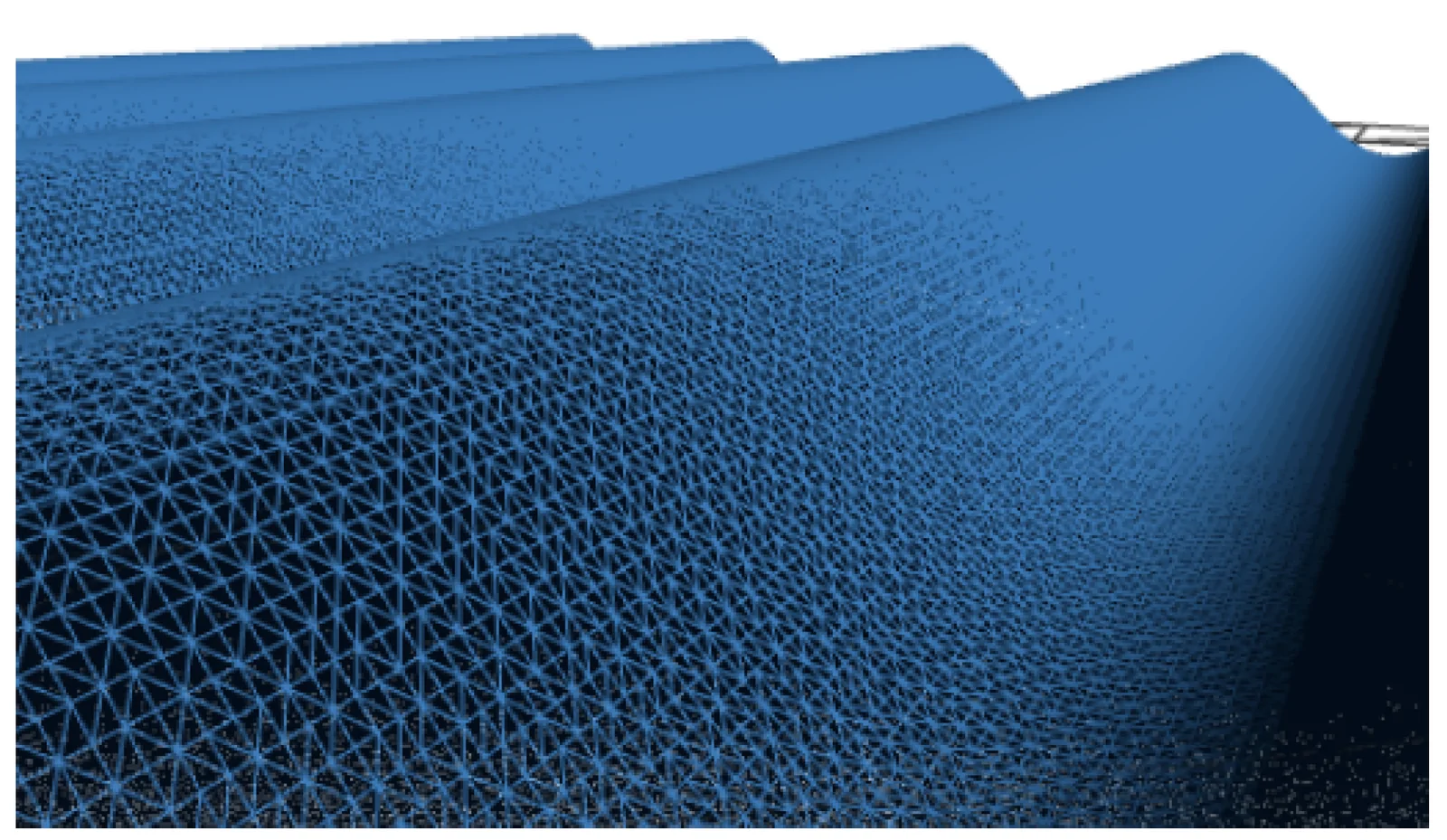
Example wireframe of the STL file for texture 1 generated by Equation (1). We used step size 0.1, which gave more than high enough resolution given the constraints of nozzle size of the printers.

All the models were converted to file type STL as blocks to be 3D printed. These models were all constrained to be the same maximum height of 7 mm to reduce experimental variation by allowing the sensor to be lowered to the same position for each experiment. When generating the patterns shown in Figure 1, we used a step size of 0.1, which converts to 0.53 mm per step, with 189 points in the x-direction and 201 points in the y-direction so that the blocks are not perfectly square, giving blocks of dimension 23.3 mm × 30 mm. This was done to prevent human error and ensure that the blocks fit in the testing rig only one way. The points form the vertices of the triangulated STL mesh, as seen in Figure 2. The smaller the step size, the larger the STL file and the higher the geometric fidelity of the exported model. The step size was selected such that the resulting mesh discretisation error (e.g., chordal deviation) remained below the nominal manufacturing resolution of the printers listed in Table 1. Consequently, any observed limitations in surface smoothness are attributable to the manufacturing process rather than the STL export. For FDM printing, increasing the STL resolution beyond this point provides no practical benefit as the achievable geometry is ultimately constrained by the nozzle diameter and layer height. The same resolution constraints do not apply to the resin printer. However, from prototype prints, a step size of 0.1 proved to be smooth upon visual inspection, so we did not need to increase resolution.

### 2.3. Tactile Dataset Printing and Collection

#### 2.3.1. Experimental Setup

**Figure 3:**
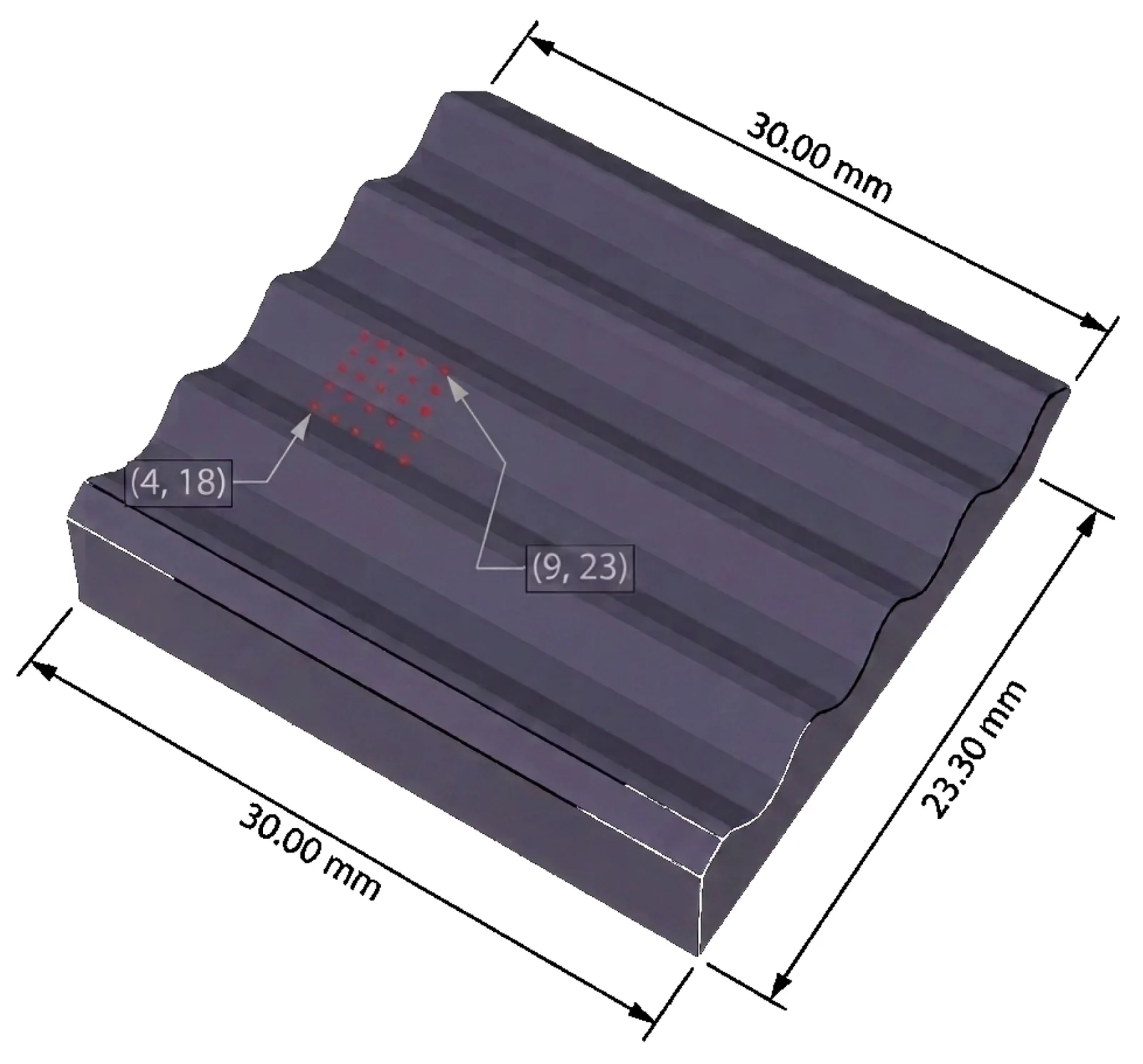
A diagram to show an example block, and the positions that the readings have been taken from in the 5 × 5 grid. Each red point is where the lowest point of the TacTip soft body will press.

**Figure 4:**
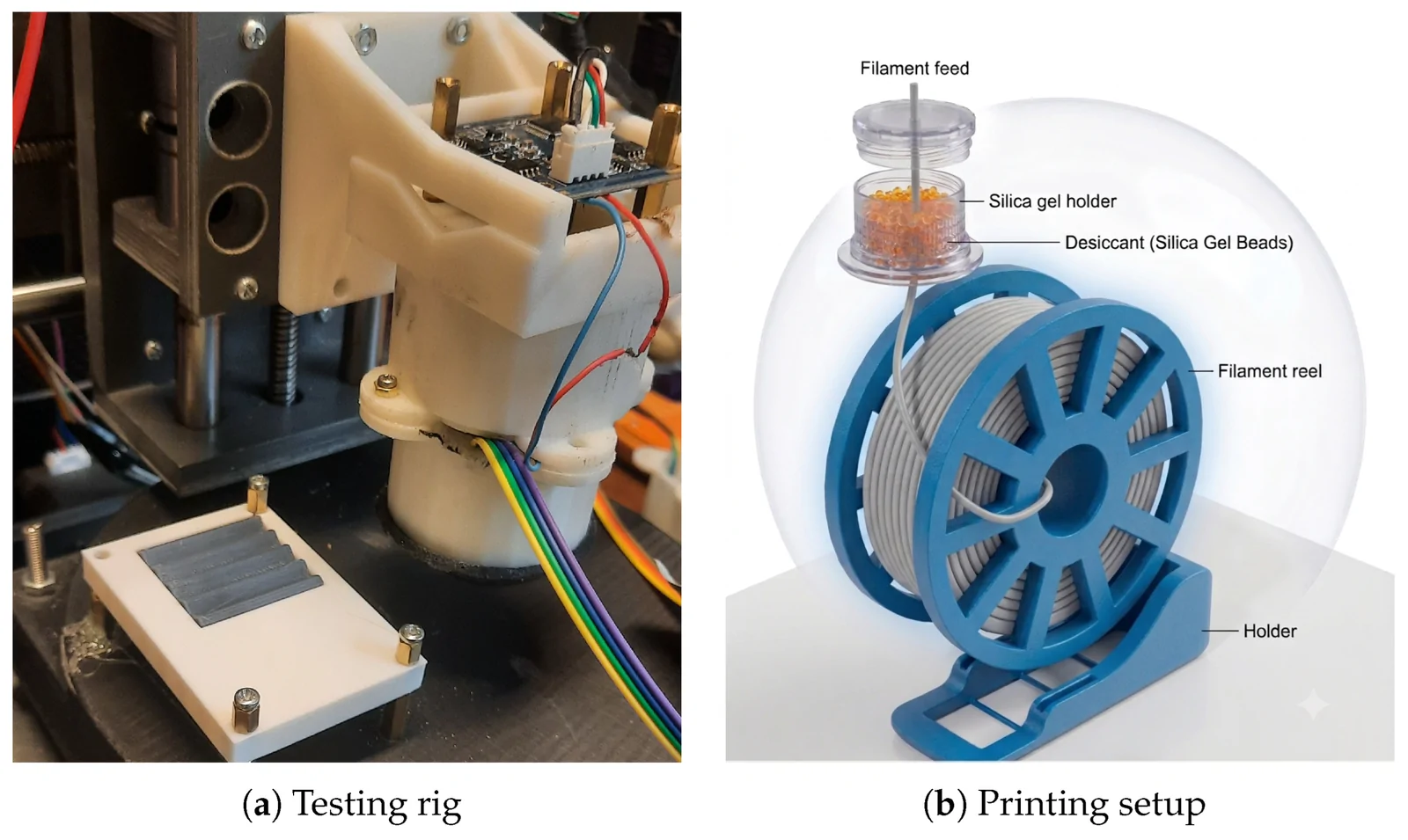
(**a**) Rig with new texture housing unit attached. Each texture slots into the unit, allowing for the sensor to be pressed. (**b**) Render of the reel housing unit for storing the filament in a non-humid environment. For visualisation, the outer housing is not shown, but the reel is inside an airtight container.

To gather the dataset, we used a TacTip tactile sensor [1] with 133 × 1 mm optical markers pressed onto a grid of positions across the texture. Figure 3 shows the points of contact for the TacTip sensor. Each sensor is pressed twice on the contact points. A texture housing unit (seen in Figure 4a) was 3D printed to hold each of the texture blocks seen in Figure 1. The testing rig pressed the sensor over each texture by pressing the sensor in a rectangular grid taking snapshots of the tactile sensation. The x position of the centre of the TacTip was moved to 5 equally spaced positions between 4 mm and 9 mm, and the corresponding y position was moved to 5 equally-spaced 1 mm positions between 18 mm and 23 mm, giving 25 points across the texture. This was repeated twice, giving a dataset of 50 images. Previous experiments have used varying force levels [10,26,27]). However, in [10], classification was robust across a range of forces. Therefore, we only used one sensor contact force for our dataset: approximately 1.9 N.

**Figure 5:**
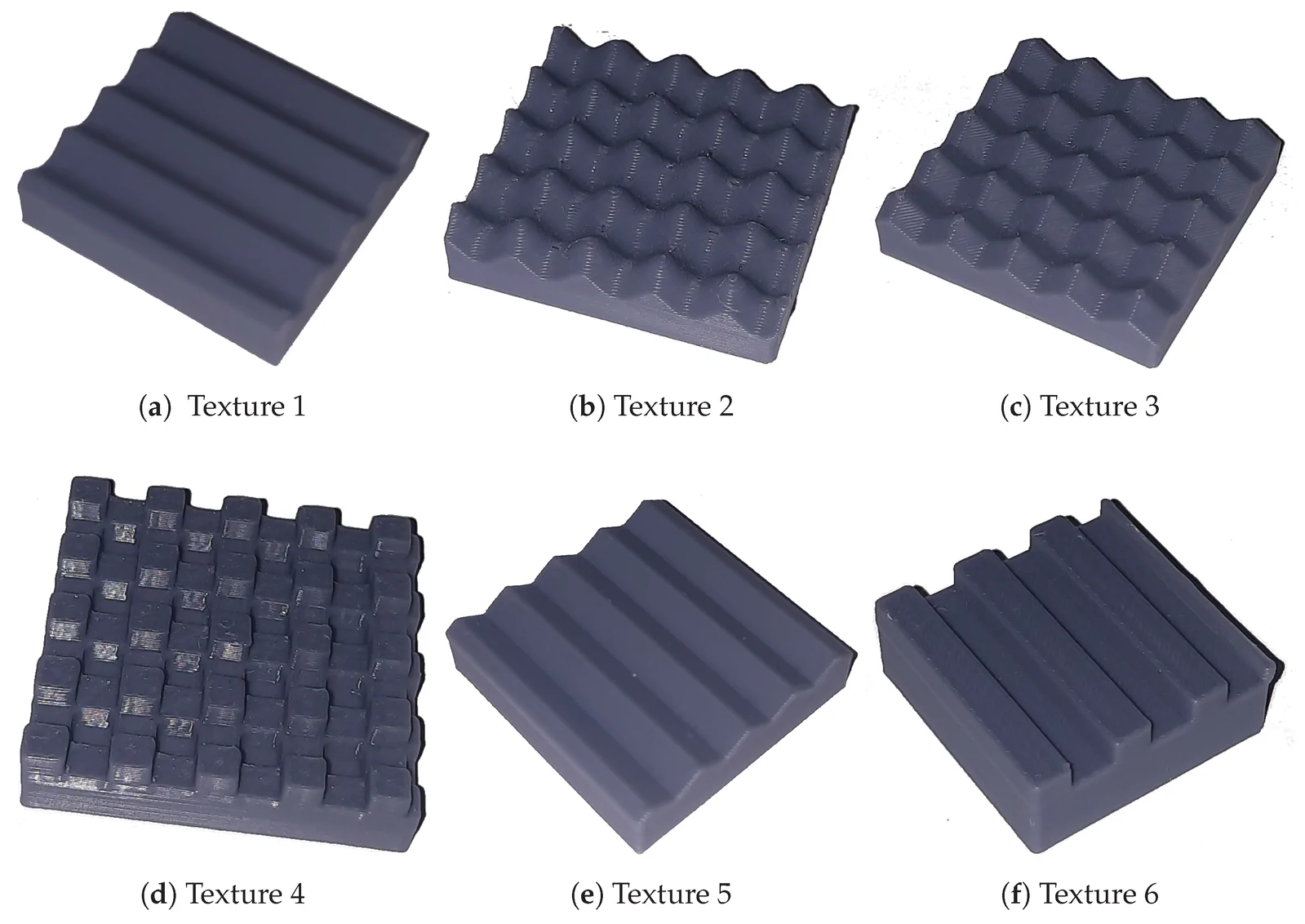
The 3D-printable textures printed in PLA- on a Bambu printer.

Figure 5 shows an example of one batch of the Bambu prints.

#### 2.3.2. 3D Printers

We selected four printers, chosen for popularity and access: An Ender-3 printer; a Creality Ender V3-SE printer; a BambuLab P1P printer; and a Formlabs Form 3 resin printer using Resin Grey v4.1. Each printer type was tested with a single representative printer of that model, as we did not have access to more than one of each type. The printer specifications are outlined in Table 1. Resin printers are generally more expensive but yield better quality for small detail. Some labs will have different printers to these, so replicating our methods will allow researchers to see whether their printer can produce a high enough standard.

We used three different 1.75 mm filaments: Rapid PETG (Elegoo), PLA+ (eSUN), and PLA-Lite (eSUN). For the resin printer, we used Grey V4.1. According to manufacturer specifications, these filaments have a dimensional tolerance of ±0.02 mm. We chose PLA filaments as PLA is an economical standard across many 3D-printer users. PLA-Lite (which we refer to as PLA-) is cheaper than PLA+, though PLA+ is supposed to be higher quality, so we chose to explore whether this had an impact. We used PETG as it provides durability but can be trickier to print with, potentially introducing more variance unless print settings are tuned properly.

#### 2.3.3. Humidity and Environment Control

To minimise the effects of moisture absorption during filament storage, all filament spools were stored in sealed dry containers based on the *Ultimate Filament Storage* cereal box design from BambuLabs [28]. Each container incorporated dedicated desiccant compartments and an integrated hygrometer, allowing the internal humidity to be monitored while maintaining a low-moisture environment. The sealed containers reduced the exposure of hygroscopic filaments to ambient humidity between printing sessions, helping to prevent moisture-related defects such as bubbling, stringing, and reduced inter-layer adhesion. This storage approach minimises variations in print quality caused by filament moisture content, improving the repeatability and consistency of the printed components used throughout this work. The render of this container can be seen in Figure 4b. Prior to the filaments being stored in the container, they were put in a filament dryer, PLA for 8 h at 50 °C and PETG for 60 °C for 8 h. Both were then immediately placed in the containers.

In terms of temperature and humidity control during the printing process, the Form 3 resin printer operates within an enclosed and temperature-regulated environment, reducing the influence of ambient conditions on print quality. All printers were located within the same laboratory space, where humidity was managed using an air purification system. Consequently, while environmental factors cannot be entirely eliminated, the printing conditions were kept consistent across all experiments.

#### 2.3.4. Scalability and Hardware Limitations

While the benchmark geometries are saved as unitless STL files, making the underlying mathematical mesh inherently scalable to any dimension, the physical instantiation of these textures remains bounded by the minimum feature size and resolution of the specific 3D printer used. This study validates the benchmark at a single physical scale. Scaling the textures down significantly may result in the loss of high-frequency geometric features (such as wave peaks) if they fall below the printer’s manufacturing limits, while scaling up may alter the mechanical interaction dynamics with the tactile sensor.

### 2.4. Evaluation of Materials and Print Quality

We wanted to evaluate the reproducibility of textures produced by different 3D printers and whether different filament brands and types differ. If a cheaper filament, or printer, is significantly worse in quality than others, then we need to advise against using this, or find combinations of print settings mitigating these issues. We employed two methods of evaluation, one using an RGBD Intel Realsense D405 stereoscopic camera (Intel Corporation, Santa Clara, CA, USA) to assess structural differences, and the other using a Tactile sensor (TacTip) to investigate the variance in sensor response and how this variance impacted classification tasks.

#### 2.4.1. 3D Scan Evaluation

**Figure 6:**
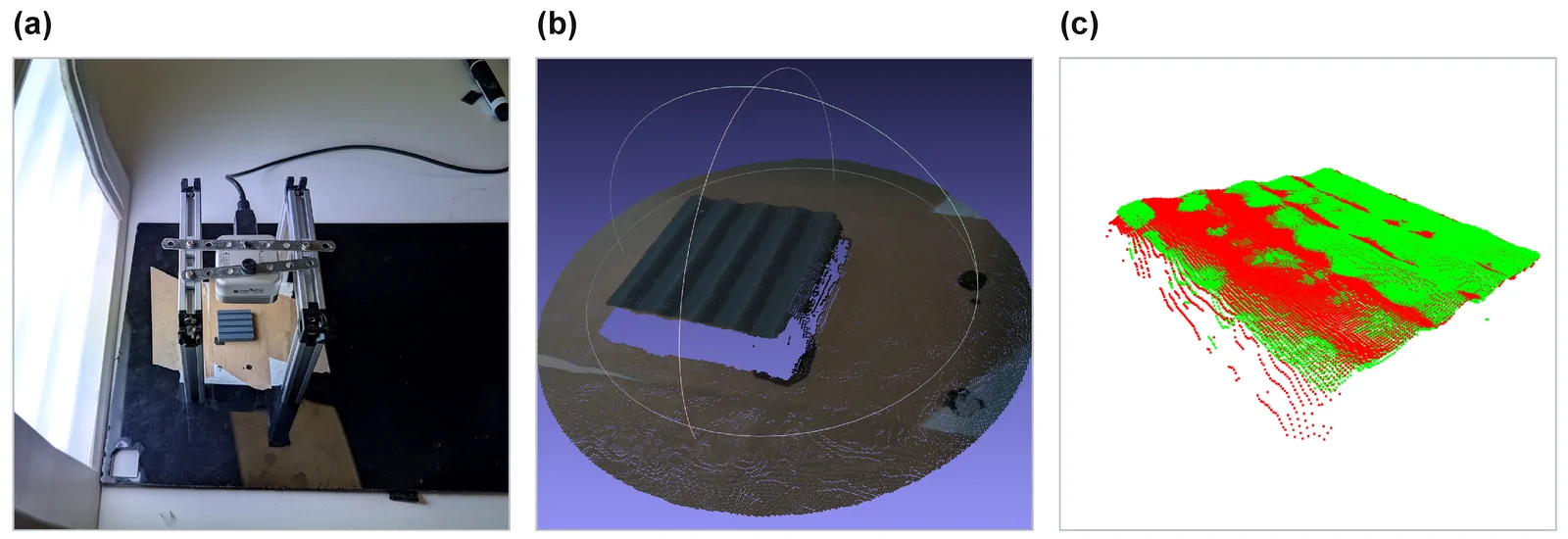
(**a**) Scanning rig for capturing 3D point clouds of the texture blocks. Top lamp not shown here for clarity. (**b**) Visualisation of captured point cloud of a texture block after cropping. (**c**) Alignment process during the comparison of two texture block. Red is one texture block scan and green is the other.

For each, printer, filament and texture combination that we investigated in detail, we made five repeat prints. We then used the RGBD Intel Realsense D405 stereoscopic camera to scan the texture blocks in 3D. The D405 is a stereoscopic depth camera with a manufacturer-stated depth precision of <1 mm at its optimal range. However, when comparing differences between repeated prints, errors can be introduced from other sources (surface reflectance, lighting, point cloud matching) that are not due to differences in the 3D texture. We therefore assessed what we term the method error (derived from repeated scans of the same block) and treat this as the effective resolution of the 3D measurement. We used Intel Realsense viewer software to record at a resolution of 848 × 480 and exported the scans to PLY format (further software recording parameters are provided in the Appendix A within Table A1 and Table A2). We recorded the texture blocks from a top-down perspective at a range of 8.5 cm, with supplementary lighting provided by lamps on the side and top of the scanning rig (setup shown in Figure 6a). These scans were static from only one perspective, so aspects of the texture blocks side or underside were not captured.

We saved each scan as a coloured 3D point cloud (Figure 6b), then filtered the point cloud so that only points from the texture remained (Figure 6c). Specifically, each scan was preprocessed to remove scan noise by finding the largest cluster of points (which would be what the camera captures directly in front of it). The centroid point of this cluster was calculated, and a 50 mm radius was cropped (as seen in Figure 6b). Finally, we removed everything under the z-axis threshold of 2 mm from the z-axis centroid to only keep the top of the point clouds, as can be seen in the two point clouds in Figure 6c.

The processed point clouds were aligned using an iterative closest point [29] (ICP) registration algorithm. Floor regions were first removed through RANSAC plane segmentation using a distance threshold of 2 mm. A multi-stage point-to-point ICP procedure was then applied using progressively smaller voxel sizes and correspondence thresholds. This coarse-to-fine registration approach initially corrected larger positional offsets before refining the alignment to achieve a high-resolution fit and resulted in a transformation matrix $T^{*}$ as follows:

(6) $$T^{*}=\arg\min_{T}\sum_{i}{∥p_{i}^{\mathrm{target}}-T\left(p_{i}^{\mathrm{source}}\right)∥}^{2}$$

where $p_{i}$ is the position of a point in either the target or source point cloud and *T* is a candidate transformation. Because this algorithm minimises the distance between point clouds, the final transformation matrix, $T^{*}$, obtained from ICP, is applied to the source point cloud, and the mean distance, d, averaged across all points in the point cloud, is used as the distance between two repetitions of a printed texture.

To calculate how much variance is introduced for a particular texture by the printer–filament combinations specified in Table 2, we compare the mean distance between each combination of repeats (i.e., repeat 1, compared to repeats 2 to 5, repeat 2 compared to repeats 1 and 3 to 5, etc.). As the ICP algorithm is asymmetric in how it treats target and source, this gives 20 comparisons in total for each texture and printer–filament combination. We call this the *raw error* and present the mean and standard deviation across the 20 comparisons in Table 2.

As well as the raw error, we also report what we call the *method error*. This is calculated using the procedure above, but the comparisons are made from five scans of the **same** block, for all six textures and each printer–filament combination. This gives 20 comparisons for each texture and printer–filament combination and we again present the mean and standard deviation in Table 2. The resulting differences show how much the points differ due to LED lighting, print surface reflection, camera noise, and point cloud matching. These variations are referred to as the noise floor.

#### 2.4.2. Tactile Image Evaluation

To assess how much images from the TacTip varied, we calculated the differences between images gathered from a TacTip sensor pressed into the five repeat prints (from each texture, printer–filament combination), as pressing the sensor on the same part of the block should result in similar sensor readings. For each print, we pressed the TacTip twice on 25 positions giving 50 images per print. We then calculated the mean-squared difference between all pixels:

(7) $$Imdiff\left(x,y,p,q,a,b\right)=\frac{\sum_{i=0}^{n-1}{\left(Im{\left(x,y,press_{p},repeat_{a}\right)}_{i}-Im{\left(x,y,press_{q},repeat_{b}\right)}_{i}\right)}^{2}}{n}$$

where $Im_{i}$ is the *i*th pixel in an image and we calculate the mean across the *n* pixels. For the image, x and y are coordinates of the positions, $press_{p}$ denotes which of the two presses is used (so p∈1,2) and $repeat_{a}$ is which repeat of the block is compared with a∈1:5. To calculate the mean difference between repeats, we calculated Imdiff using Equation (7) for unique combinations of repeats (i.e., repeat 1 vs. repeats 2 to 5; repeat 2 vs. repeats 3 to 5, etc., giving 10 combinations for five repeats and 6 combinations for four repeats) for each of the 25 positions (x,y) and each of the two presses (so p=q in Equation (7)). This gave 10×25×2=500 differences when there are five repeats and 6×25×2=300 when there are only four.

To capture the noise introduced by fluctuations in LEDs, positioning, etc., we also calculated a method error for the tactile image comparison. To do this, we calculated the differences between press one and press two for a given position for the same block, i.e.,: Imdiff(x,y,1,2,a,a) where a∈1:5. This resulted in 25×5=125 differences for five repeats and 100 differences for four repeats. We also gathered a recording of the TacTip stationary, not pressed on anything, to assess the component of error from LED fluctuation. We calculated the MSE between 136 frames of recording to calculate the background noise, as in Equation (7), between each frame. The average fluctuation was 3.67, which sits far below the method error differences shown in Section 3.3.

We tried to obtain readings from five repeats for each texture and printer–filament combination. However, there were some corruptions in the dataset, where LED power fluctuated and the image was too dark to see anything. Corruption occurred in two samples (Ender 3 using PLA+, referred to as Ender-3 in Table 2, Table 3 and Table 4, Texture 6, repeat 1; Resin, Texture 1, repeat 1). This meant that these texture and printer combinations had one less set of Tactile images, which reduced the overall dataset by 100 images.

### 2.5. Benchmark Reproduction Workflow

To encourage reproducibility and facilitate future tactile sensing research, all resources used to construct the benchmark are publicly available through the accompanying GitHub repository. The repository contains the complete pipeline for generating printable texture samples, producing standardised printer-ready files, and reproducing the dataset collection process.

The repository is organised into three primary components. First, a library of printable texture models is provided (3DD1), including procedurally generated surface geometries. Secondly, the repository includes scripts for generating custom textures from user-defined parameters (3DD2), allowing researchers to extend the benchmark with additional tactile patterns while maintaining consistent geometric constraints. Finally, standardised printing profiles are supplied to support benchmark samples being fabricated consistently across compatible material extrusion 3D printers (3DD3). Naming conventions are outlined in Appendix A.

The recommended reproduction workflow consists of four stages. First, users generate or select a texture model from the provided library [30]. Secondly, the model is sliced using the supplied printer profiles, ensuring identical print settings such as layer height, nozzle diameter, infill, and print orientation. Thirdly, the printed sample is fabricated using the specified filament materials and environmental conditions. Finally, the completed sample can be incorporated directly into the tactile data collection pipeline described in this work.

As both the source models and the complete fabrication pipeline have been released, researchers are able not only to reproduce the benchmark presented here but also to generate additional textures that remain compatible with the benchmark methodology. This enables direct comparison between future tactile sensing approaches while promoting consistency across independently collected datasets.

## 3. Results

### 3.1. Variability Across Printer and Filaments

To assess how much prints from different printers and filament combinations varied, we first used visual inspection to judge the quality of a print, particularly for “stringing”. Stringing is when the printer’s nozzle leaks a small amount of molten filament while moving between separate parts or sections, leaving thin strands of plastic connecting them. If, from a human perspective, quality varies, this likely means the print quality has high variance.

**Figure 7:**
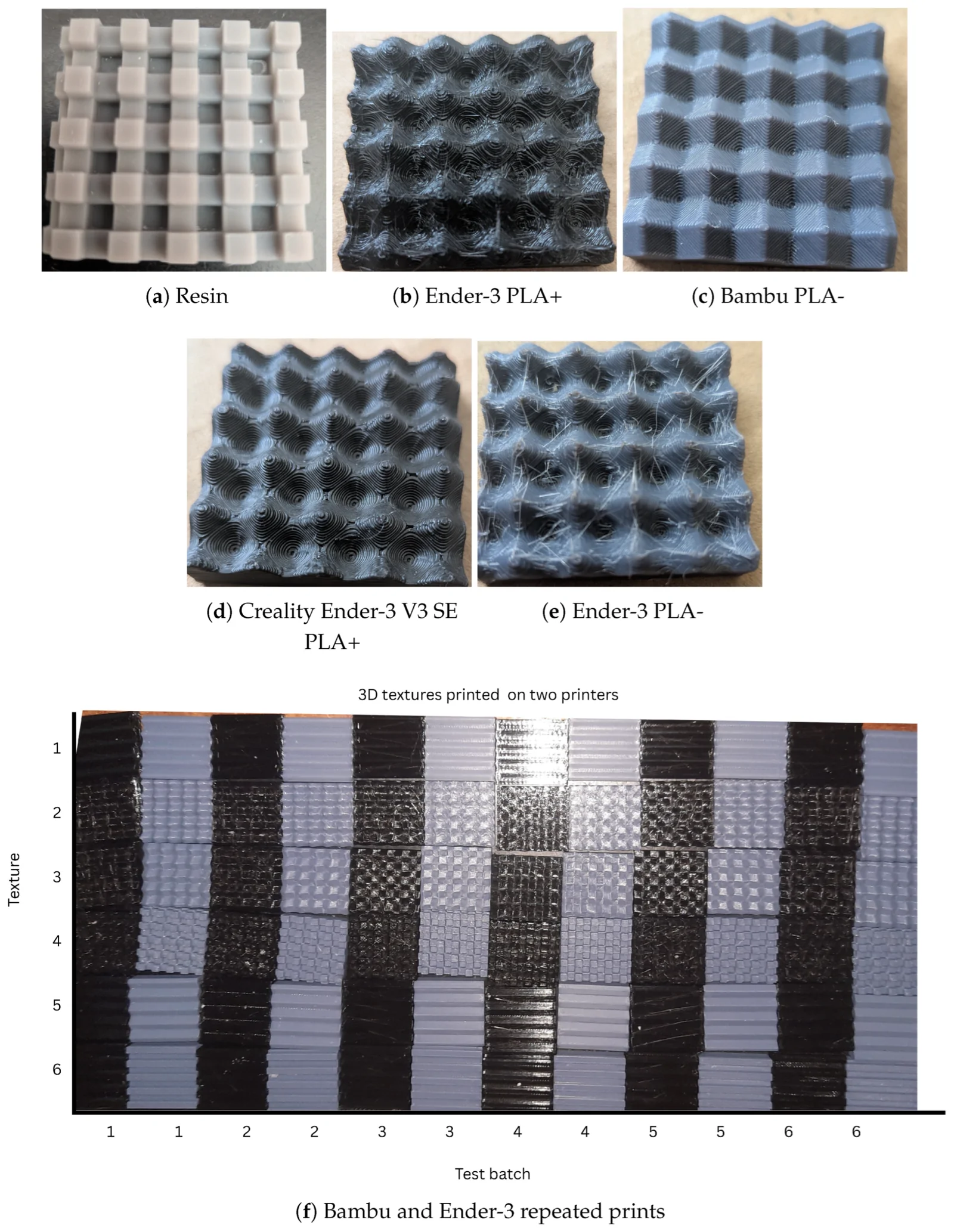
Examples of the prints. The black filament (close up shown in (**b**,**d**)) is PLA+ printed with the Ender-3 (**b**) and the Creality Ender-3 V3 SE, whereas (**c**) shows the Bambu prints and (**e**) Ender-3 both with PLA-, which is grey. The Ender-3 and Creality Ender-3 V3 SE had a lot of stringing on all filaments used, and ridges were more visible. It is clear that there are no stringing effects on the resin printer (**a**). In (**f**), we show an example of the six textures printed five times on the Bambu with PLA- (grey) and Ender-3 with PLA+ (black).

When we inspected the prints, some had more plastic webbing/string between blocks, especially prints produced with the Ender-3 with different filaments (seen in Figure 7b,e where there are thin plastic lines between the peaks); however, these did not affect the surface of the block. More importantly, some of the peaks were more defined than other peaks on the same print. The sensor for dataset gathering comes from the top and presses down so stringing between peaks is unlikely to be picked up at this scale. However, differences in peak heights, and well-defined summits, will be.

We next selected a subset of printers that spans the print-quality spectrum (as judged visually) for more detailed evaluation: the Bambu Lab paired with the lower-cost PLA- filament (henceforth referred to as ‘Bambu’), the Ender-3 paired with PLA+ (henceforth referred to as ‘Ender-3’), and the resin printer with grey V4.1 (henceforth ‘Resin’) as a visually low-variance baseline. The Creality Ender-3 V3 SE was excluded from the extremes-spanning subset as we already had higher-end and lower-end printers. This selection enables detailed comparison of print variability across different levels of printer capability and material quality.

To assess the quality of prints, we generated five prints of each texture from each of these printer–filament combinations. We first assessed how much prints varied based on 3D scans of the texture blocks and then assessed how much images from a commonly used Tactile sensor differ between repeats. Finally, to assess whether these differences were functionally important, we assessed how well texture classification models performed when trained on one repeat and tested on the others.

### 3.2. 3D Scan

To assess print variability, we made five prints of each of the six textures for each of the three selected printer–filament combinations. We measured the 3D structure of the blocks using an RGBD camera and assessed the difference between repeated prints as described in Section 2.4.1. To account for scan error, we placed the same block in the scanner for each texture and scanned it five times for each of the six textures. We then compared this *method error* to the differences between repeats to see if the difference between prints was above measurement noise.

The average point cloud deviations for each manufacturing method are presented in Table 2. The raw distance errors are the differences between scans of repeated prints of a texture after Iterative Closest Point (ICP) alignment. The method error are differences between scans of the same block, providing an empirical baseline of the overall repeatability error. As can be seen, the Ender-3 is the worst performing overall of our sample of printers with higher difference between print batches than the Bambu and Resin (aside from Texture 2 for the resin printer). As the method errors are broadly similar between printers, the larger Ender-3 differences are due to print quality, as they are mostly more than twice the size of the method errors. Aside from Texture 2, the Bambu and Resin have similar differences, with the Bambu superior on four textures, and Resin slightly better on two. However, differences between repeated prints lie near the noise floor (i.e., method error differences), so it is unclear how significant they are. That is, because the differences between the Bambu and Resin repeats are similar to the method error for these printers, these differences cannot be reliably resolved by this measurement.

Note that this analysis compares prints with one another and does not compare them with the nominal STL geometry. While it therefore measures repeatability, two prints could agree closely while both deviating from the STL design. To rule out this issue, we also compared the data from the point clouds from the ‘ideal’ pattern of the STL. To do this, we used the same method as for the cross-repeat difference, which is based on ICP alignment. While this introduces some noise, this should be the same for all printers (as was seen in Table 2). The differences between prints and the STL are presented in Table 3. As with the differences between repeats (Table 2), the Ender configuration is the most different from the STL, while the Bambu and Resin configurations perform about equally.

Because of the difficulties in disambiguating differences from noise, and to assess how much these differences affect the behaviour of a typical sensor, in the next section we assess how much the response of a tactile sensor varies across repeated texture prints.

### 3.3. Tactile Image

**Figure 8:**
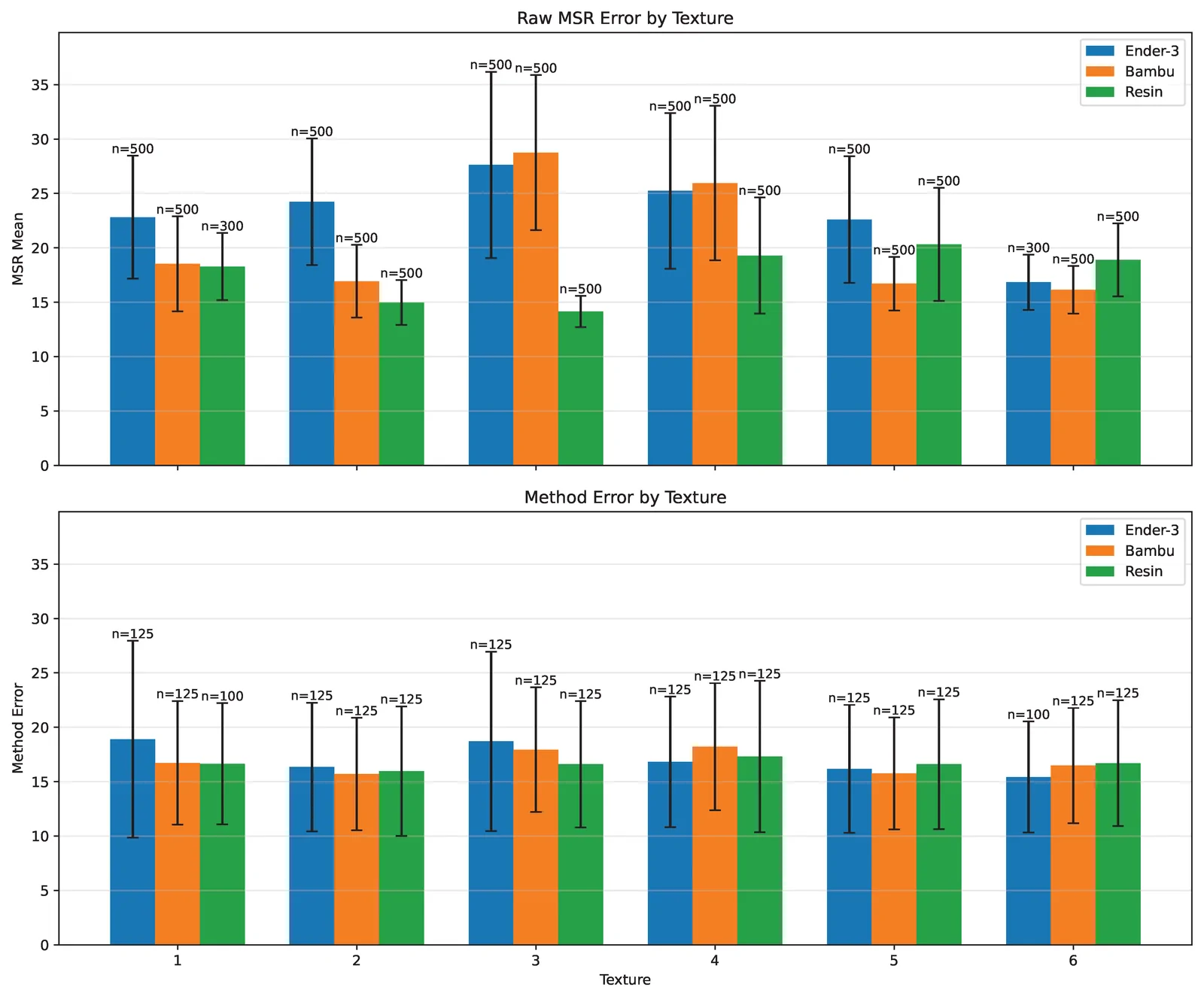
The top chart shows averaged image difference for three different printers across each texture with error bars showing the standard deviations of pixel difference. Data are collected from n = 300–500 image differences for each texture (with the amount indicated above the error bar). Note that these image differences are not independent as they are taken from differences between only four or five independently fabricated blocks. The large number of image differences is because we calculate differences from 25 positions, two presses, and compare between unique combinations of the blocks. The bottom chart shows the mean and standard deviation of the method error, where repeat presses of the same block are compared. This is made up of n = 100–125 image differences, with n shown above the error bar. As for the top chart, the data is not independent as there are 25 differences—one per position—for each independent block. Both charts include data for the Ender-3 using PLA+ (blue), Bambu Lab using PLA- (orange), and FormLabs Resin printer using grey V4.1 (green).

To assess variability between tactile images, we collected TacTip readings from 25 positions shown in Figure 3, taking two readings per position, across each repeat texture. For each texture and printer–filament combination, we then calculated the mean pixel-level difference between repeat trials for each of the 25 positions between unique combinations of repeats as in Equation (7). We refer to this as the raw error. To assess a method error, instead of calculating the differences between repeats, we calculated the difference between each of the two presses on the same block for each position. The results for each texture and grouped by printer–filament combination are shown in Figure 8. As seen in the differences from 3D scans, while all printers had similar method errors, raw differences varied by texture and printer. Of the filament printers, Bambu with PLA+ had a lower difference between repeated prints than the Ender-3 with PLA- for four of the six textures, but a slightly higher difference for textures 3 and 4. The resin configuration had the most consistent difference across textures and the lowest overall difference for textures 1 to 4. Overall, however, as with the 3D measurements, there was no outright ‘winner’ but a more general pattern of the Ender-3 with PLA- being slightly worse than the other two configurations.

**Figure 9:**
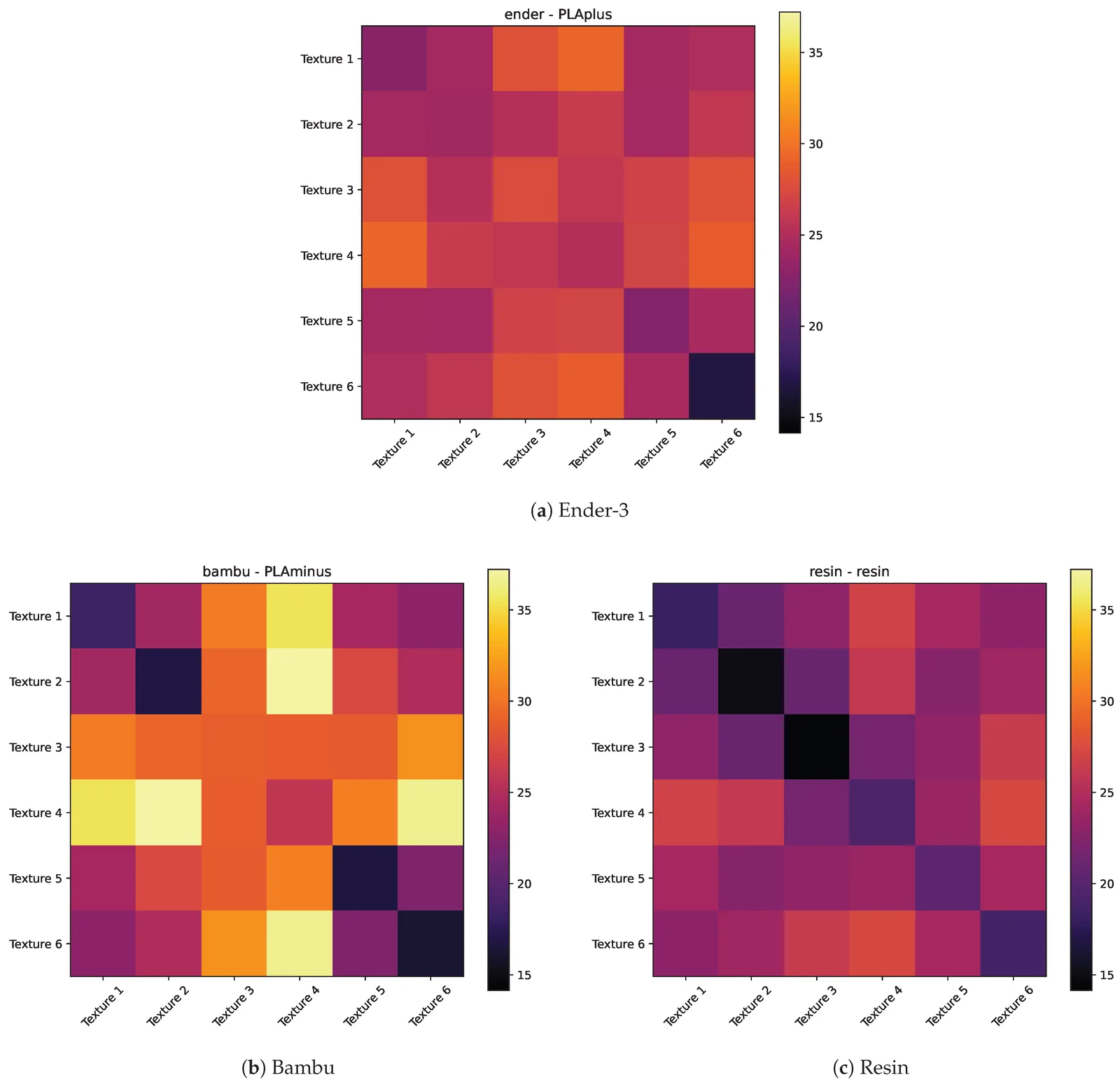
The mean difference between pixels for each texture, compared between each of the textures. This table contains data for the Ender-3 using PLA+, Bambu Lab using PLA-, and FormLabs Resin printer using grey V4.1. Differences are generated between the same press, position, and repeat but for different textures of the same print configuration (i.e., press A, from position B and repeat C of texture 1, is compared to press A, position B and repeat C of each of textures 2 to 6). The differences are then averaged over the 2 × 25 × 5 = 250 differences (apart from row/column 6 for the Ender-3, and row/column 1 for Resin, which have one fewer repeat, so 200 differences).

As we are going to use this data for classification, we also examined the average differences between different textures (Figure 9), as this can tell us whether the differences in repeated prints would mask differences between textures. The Ender-3 had the most overlap between texture images (as judged by the difference scores), reflecting the high differences between print batches of the same textures (aside from texture 6). The Bambu had a clearer trend in lower differences for the same texture, though textures 3 and 4 had high differences throughout, with texture 3 looking especially complex. These features would be harder to classify. The resin printer had the clearest picture overall with the diagonal elements the lowest and then rising differences off-diagonal. The Bambu additionally had a lot of crossover between textures 1 and 2, and textures 5 and 6, where we saw two blocks of low difference. On closer inspection, both the Ender-3 and Resin printers show similar, if less obvious, lower difference for these patterns. This reflects the similarities between textures 1 and 2 and textures 5 and 6 from a tactile sensor’s perspective (which emphasises the higher points of the patterns).

### 3.4. Effect of Printer Variability on Texture Classification

In the previous section, we saw that different printers differ in print variability. We next wanted to know whether this variance has an effect on classification. To do this, we trained classifiers to classify textures from different printer and filament combinations. To mitigate time-varying sensor artifacts, such as marker tracking noise or transient illumination fluctuations, two independent readings were recorded at each relative texture position. Furthermore, systematic sensor behaviours (e.g., elastomer deformation and hysteresis) were kept constant across all trials by utilising a single TacTip sensor profile under identical robotic displacement constraints, so that variations in the signal directly reflect physical manufacturing defects. To make sure our results were not due to a particular classifier, we trained both a Random Forest Classifier and a feedforward Artificial Neural Network (ANN) (classifiers chosen following our previous work on tactile texture classification [10]). A Random Forest Classifier was trained using the default scikit-learn hyperparameters. The classifier comprised 50 decision trees, using the Gini impurity criterion for splitting. Trees were allowed to grow without a predefined maximum depth, while $\sqrt{n}$ features were considered at each split. Bootstrap sampling was enabled, with the remaining parameters retained at their scikit-learn default values. This was in line with previous experiments [10]. The ANN used the same setup as existing TacTip classification [10], namely a single hidden layer of size 50 and an output layer of 6. To train the ANN, we used cross-entropy loss with the Adam optimizer at a learning rate of 0.001. Training took place over 1500 epochs. These classifiers were trained from randomly split data, and random initialisation states of the classifier; therefore, our results shown are averaged over 20 trials.

The dataset consisted of all five repeats of each of the six textures produced using all printer–filament selections. As there are two presses at 25 positions, this resulted in 2 (presses) × 25 (positions) × 5 (repeats) × 6 (textures) × 3 (printer–filaments) = 4500 images. Removing the two corrupted samples left a dataset of 4400 images. To reduce memory requirements, all images were converted to greyscale and downsampled to 40% of their original resolution, a preprocessing step previously shown not to affect classification performance [10]. After preliminary investigation, we also decided to use PCA to convert the dataset to a smaller dimensionality thus removing noise and highlighting useful features for the classifiers. PCA was also applied to the training split of the dataset to convert the images to lower dimensions. For the augmentation experiments, we used 25 principal components because it was faster and results showed good accuracy; however, for the full cross-batch experiments, we used 300 components and extracted the components from training data after augmentation. The principal components were then used as input to the classifiers. In each case, the test split was then transformed using the fitted PCA.

We applied augmentation to the training datasets to account for LED noise and global lighting variation. Specifically, for each image, we added Gaussian noise with mean 0 and standard deviation 5 to each pixel, making one new image (‘Noise’ augmentation). We also added/subtracted 10 to each pixel to ‘vary’ the lighting, resulting in two new images (‘Brightness’ augmentation). To see if these augmentations were useful, we ran a preliminary test using only the RFC and with only 25 PCA components. Because we wanted to assess whether the augmentation helped with variability across prints, we trained the RFC on all the data from batch 1 and tested it on batch 2, with the accuracy being the performance on the test set. The results of the preliminary ablation study are shown in Table 4. Brightness did not help accuracy; therefore, we continued with noise injection only.

**Figure 10:**
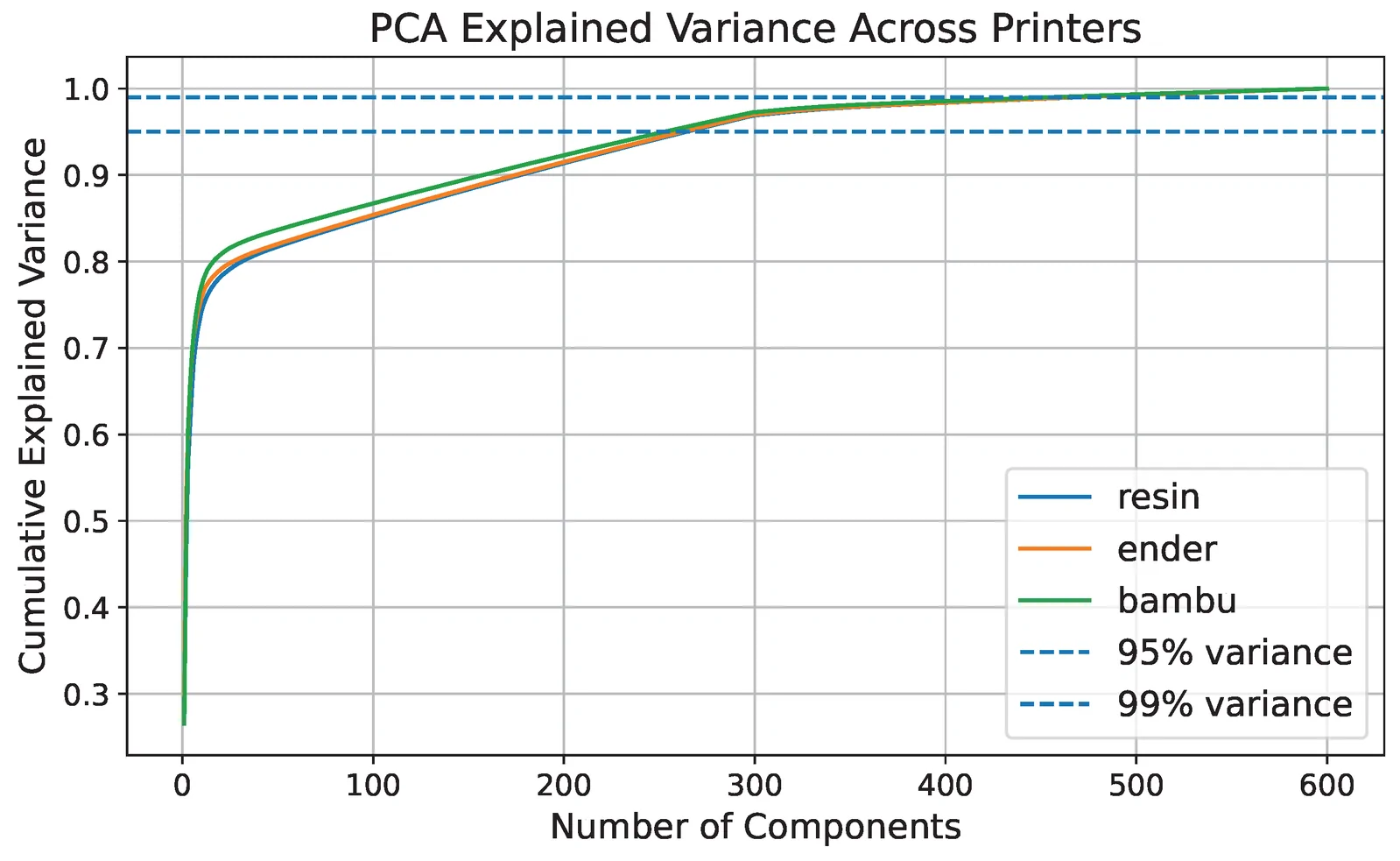
Cumulative explained variance of principal component analysis (PCA) for tactile images. PCA variance from one augmented batch of data from the Bambu, resin, and Ender-3 printers. The cumulative variance is plotted against the number of principal components required to represent each dataset. Dashed horizontal lines indicate the 95% and 99% explained variance thresholds. Differences between the curves reflect the intrinsic variability of tactile features produced by each printing process, with datasets requiring more principal components exhibiting greater variation in surface characteristics.

After establishing which augmentation worked well in our preliminary experiments shown in Table 4, we then tested both the ANN and RFC classifiers with the full dataset for a final cross-batch experiment. Analysis of the amount of variance explained by PCA components (Figure 10) shows that the amount of variance explained plateaued around 300 components, and thus was chosen to keep the data dimensionality low without dropping meaning in the data. To assess the impact of manufacturing repeatability, each classifier was trained/tested using a single batch of prints from a specific printer–filament configuration, but then additionally evaluated by testing the trained classifier on the other four datasets, which are completely unseen to the classifier training. Each batch comprises 50 images per texture, so with noise augmentation doubling the number of images, gives 600 images per printer–filament batch across the six textures. We used a 70% training/test split. This resulted in 420 training samples and 180 same-batch test samples. After training, we tested the classifiers on the remaining four batches of data (without data augmentation), making 1150 images for Ender-3 and Resin and 1200 for the Bambu (50 images/block × 6 textures × 4 repeats = 1200; one block for each of Ender-3 and Resin was corrupt so losing 50 images one) × 3 (printer–filament combinations). Accuracy is referred to as the unseen print accuracy. Results are shown across 20 runs in Table 5, where we report classifier performance within-batch (train and test accuracy—the latter being the accuracy on the test set) and cross-batch generalisation (unseen print accuracy).

For the RFC, train and test results were near perfect, meaning it was hard to distinguish between printers. However, as can be seen in the third column of Table 5, the resin printer achieved the best performance on unseen print batches, followed by the Bambu, with the worst results coming from the Ender-3. These results reflect the differences in 3D scans and tactile images and so we assume that the increased performance for the Resin is due to the greater repeatability between prints. While classifiers might be partly exploiting print-specific manufacturing artifacts, such as stringing residue or layer-line patterns, rather than the intended texture geometry alone, the fact that the ordering of results reflects the ordering seen in differences between tactile images suggests that repeatability is the main factor. The results for the ANN were worse than the RFC for all cases (aside from the unseen accuracy on the Ender-3). However, the ordering of the unseen accuracy—with the Ender-3 much below the two other printers—again reflects the metrological data, showing that our results are not due to a particularity of the RFC.

**Figure 11:**
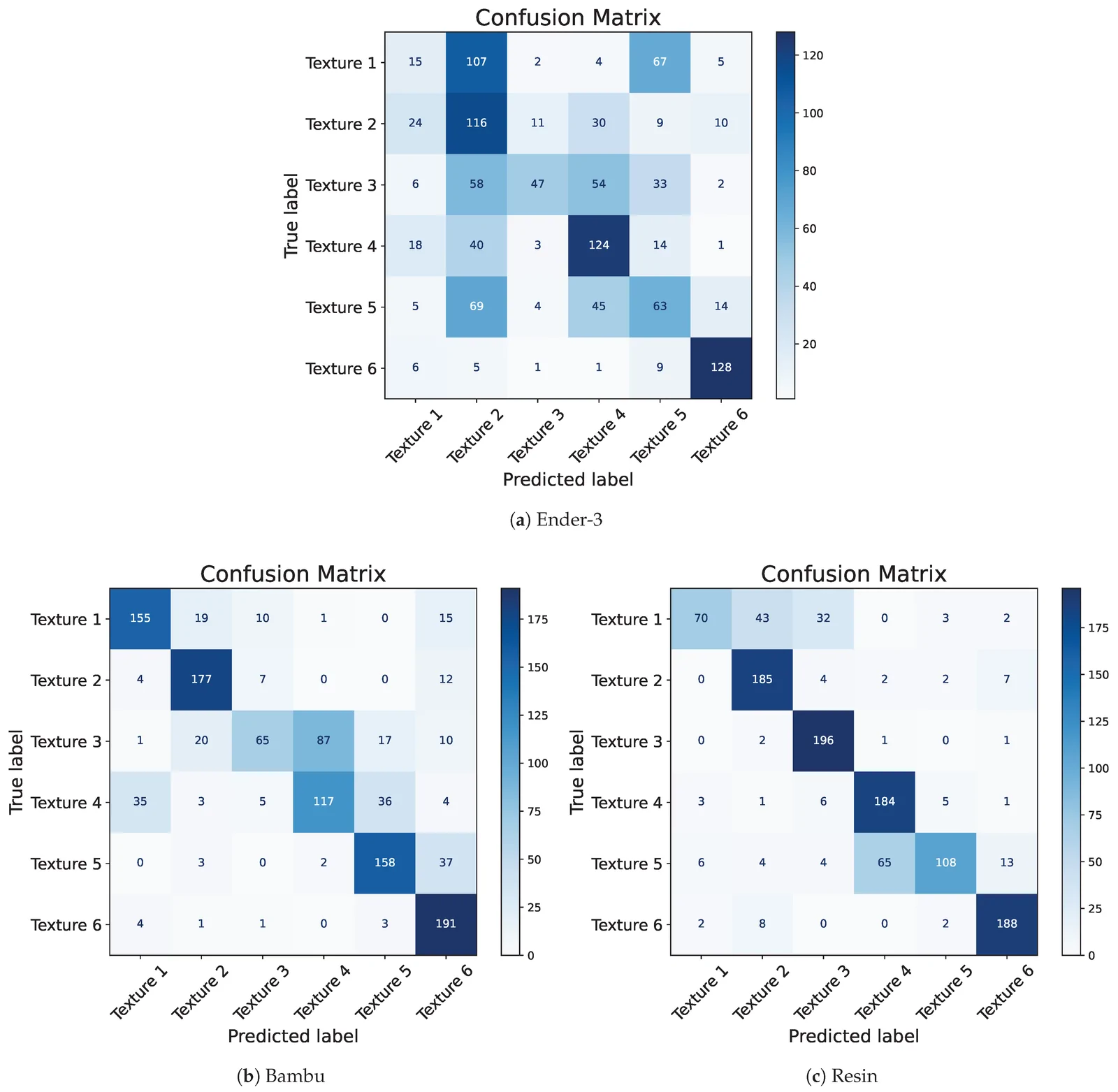
Confusion matrix for the Random Forest Classifier trained on (**a**) the Ender-3 dataset, (**b**) the Bambu dataset, and (**c**) the Resin dataset. All evaluated on unseen other prints. Performance suggests that the benchmark dataset contains texture features that are consistent across manufacturing conditions while remaining sufficiently challenging to assess classifier robustness.

When examining the confusion matrices of the Random Forest Classifier across the different printing platforms (Figure 11), the majority of the tactile profiles are correctly classified. However the systematic errors reveal insights into the physical limits of the tactile sensing pipeline.

Incorrect classifications consistently revolve around specific geometric similarities. For instance, Texture 1 is frequently misclassified as 2 and 3. In the Ender-3 and Bambu confusion matrices, textures 2 and 3 are misclassified, which is likely because they are generated from similar spatial Equations (see Figure 5 for a visual reference). Crucially, a similar cross-talk occurs between Textures 1 and 5 within the Ender-3, and there is a large bias towards Texture 2. Interestingly, this is a one-way relationship of misclassification, showing learned biases between similar patterns. Some similar relationships can be seen in Figure 9, where there is structural similarity between specific textures.

As illustrated in the Ender-3 confusion matrix (Figure 11a), the classification accuracy for Texture 1 degrades to 7.5%, with a notable 53.5% of its data mass leaking directly into Texture 2. This performance drop could be because the ultra-fine micro-geometrical traits of Texture 1 sit near the operational noise floor of the tactile sensor. On coarser extrusion systems like the Ender-3, these faint geometric signals could be masked by macro-scale manufacturing noise (such as layer shifts and extrusion variations), causing the classifier to confuse it with the more pronounced features of Texture 2. Indeed, we see this in the structural similarity plot (Figure 9a).

Conversely, the much cleaner separation of Texture 1 on the other configurations indicates that when manufacturing artifacts are minimised, the tactile sensor’s physical compliance can successfully extract the underlying texture signature. This contrast highlights that cross-print generalisation is heavily influenced by printer–filament choice, where manufacturing inconsistencies act as an undesirable confounding variable.

As a final note, consider the very high accuracies, which result in quite clean confusion matrices compared to the tactile image differences seen in Figure 9. This shows that the contact-based tactile pipeline successfully extracts invariant structural signatures from the data.

## 4. Conclusions

This paper introduces a method for generating geometric-patterned 3D-printed rigid textures that can be reproduced across different research teams, enabling fair and consistent comparisons between sensors in texture classification tasks. Our investigations highlight that filament and printer type can influence the print-to-print consistency. From the filament-fed printer models, the Bambu with PLA- had less difference between print batches than the Ender-3 with PLA+, which also experienced more stringing and overall messier prints than the Bambu. Resin with grey v4.1 was visually the best overall and had the best classification results on cross-batch generalisation, securing an unseen cross-batch accuracy of 80.27% using a Random Forest Classifier (RFC). However, the RFC classification accuracy of the Bambu was not much worse than the Resin (73.75%) and a lot higher than the Ender-3 with PLA+. The Ender configuration had very poor classification on the unseen batches (42–43%) despite high within-batch performance, indicating that the visually evident variance between batches was functionally detrimental. In contrast, both the Resin and Bambu configurations suppress manufacturing variance. The metrological results followed a similar pattern, with Bambu and Resin configurations similar in cross-batch accuracy and both better than the Ender-3. Note that the geometric scan-based ranking (Bambu marginally ahead of Resin) and the tactile classification-based ranking (Resin marginally ahead of Bambu) disagree. Given that the method errors for both scan and tactile differences were close to the raw errors (Section 3.2), we treat these metrics as unreliable for distinguishing these two configurations.

Rather than invalidating the metrological pipeline, this highlights a physical justification for the tactile benchmarking methodology. It demonstrates that the cross-print structural variations introduced by modern additive manufacturing systems, can be so small that they sit around the resolution threshold of a 3D scan using an optical scanner. Thus, this form of non-contact metrology does not reliably separate our print repeats. Further experiments using the TacTip image difference revealed similar patterns to the 3D metrology results, with again differences between batches being similar to the measurement error (derived from two presses on the same repeat) for the Resin and, to a lesser extent, the Bambu configurations. However, the contact-based TacTip sensor successfully leverages localised physical surface compliance and skin deformation to map micro-geometrical traits. This high physical sensitivity allows the machine learning framework to extract consistent structural signatures embedded within the tactile signature, securing high cross-print accuracy on both Bambu with PLA- and resin with grey v4.1 configurations.

More importantly, we feel that the good cross-batch performance of the Bambu and Resin configurations mean that these repeats share a tactile subspace, as classifiers generalise across them (Table 5). However, one potential confounding issue is that the machine learning methods could exploit printer-configuration specific artifacts. Specifically, the higher-performing classifiers might exploit subtle print-induced artifacts that are specific to one texture in addition to the intended texture geometry. Although the 3D scanning analysis quantifies inter-print geometric repeatability, it is limited by scan noise resolution, and complicated by the fact that dark (Ender-3, black PLA+), and light (Bambu, grey PLA-) filaments could scan differently. Thus, while our analysis characterises differences in print quality, we cannot exclude the possibility that such artifacts contribute to classification performance.

One factor counting against this is that the ordering of unseen-batch accuracy follows the ordering of tactile image differences (and between texture tactile differences) as well as the 3D scan differences. Second, inspection of the difference between repeats and the STL ground truth show that the areas with the greatest errors are different between repeats (Supplemental Figure S3). Additionally, we cannot see a particular “signature” being inadvertently produced by the printer. However, deeper investigation would be needed as these plots emphasise the biggest differences only.

One way of ruling out print-specific artifacts would be to show that a classifier trained on one printer–filament configuration transfers to another. If classifiers were relying on manufacturing signatures, such as stringing residue or layer lines rather than texture geometry, transfer should fail, whereas features intrinsic to the textures should carry across. However, classifiers generalised poorly across configurations (see the Supplemental Materials). As the best overlap was between the two filament printers, crossover could be driven by similarities/differences in the material (PLA vs. resin). However the results were so poor that it is hard to say for sure. Improving cross-printer generalisation would likely require a better representational space for tactile images, which is beyond the scope of this paper.

However, even if there are artifacts from our printers, it does not invalidate the overall dataset and evaluation pipeline. Indeed, we recommend that users of the benchmark fabricate their own copies, train and evaluate within that set, and report the printer–material configuration used. Testing the benchmark across multiple printers and fabrication methods, will better isolate texture-specific features from manufacturing-induced variation.

A limitation of this study is that the evaluations were conducted using a single physical unit per printer model. Consequently, observed variations incorporate localised unit-level maintenance states alongside inherent model-level performance capabilities. Again, future cross-lab studies are required to isolate machine-specific wear from broad technology-class constraints. Finally, while this work did not explore slicer-level optimisation or printer calibration, such directions offer clear opportunities to further reduce variability.

In summary, this study provides a reproducible dataset, a methodology for producing such datasets, and a structured evaluation framework for quantifying and comparing the effects of 3D-printing parameters on texture consistency, all of which can support future work in texture analysis. Our evaluation suggests that high-resolution Resin with grey V4.1 is a highly capable baseline, but that the Bambu Lab paired with PLA+ provides a strong lower-cost alternative. Importantly, while we test a limited number of printers and filaments, our evaluation methods will allow benchmark users to compare data from their printer and filament combinations to ours.

## Figures and Tables

**Table 1:** Comparison of Ender-3 (Creality, Shenzhen, China), Creality Ender-3 V3 SE, Bambu Lab P1P printers (Shenzhen Tuozhu Technology Co., Ltd., Shenzhen, China), and Formlabs Form 3 resin printer (Formlabs Inc., Somerville, MA, USA). It is worth noting that the printers all used 0.4 nozzles, though this can be reduced further.

Spec	Ender-3	Creality Ender-3 V3 SE	Bambu P1P	Formlabs Form 3 (Resin)
Build volume	220 × 220 × 250 mm	220 × 220 × 250 mm	256 × 256 × 256 mm	145 × 145 × 185 mm
Motion system	Cartesian Bed slingers	Cartesian Bed slingers	CoreXY	na
Acceleration	≤20,000 mm/s^2^	∼2500 mm/s^2^	20,000 mm/s^2^	na
Hotend temp	up to 300 °C	up to 260 °C	up to 300 °C	na
Bed temp	≤100 °C	≤100 °C	≤100 °C	na
Extruder	Direct drive	Sprite direct drive	Direct drive, all-metal	na
Extruder aperture	0.4 mm	0.4 mm	0.4 mm	na
Leveling	Auto (varies by model)	CR Touch + strain	Auto (built-in)	na
Connectivity	Touchscreen, SD/USB	Touchscreen, SD/USB-C	App, cloud, slicer	USB

**Table 2:** Results for the difference calculations specified in Section 2.4.1 for the Ender-3 using PLA+, Bambu Lab using PLA-, and FormLabs Resin printer using grey V4.1. For each printer, we show mean ± standard deviation of the raw error in one row, and method error in the subsequent row, in units of $10^{-1}$ mm. For both raw and method errors, differences are calculated by comparing each block with every other block after ICP alignment. As the alignment method is asymmetric, this results in 5×4=20 differences for each texture and printer–filament combination for both raw and method errors, while these measure are not independent, the same procedure is applied to all combinations so serves as a fair comparator.

Printer Class	Texture 1	Texture 2	Texture 3	Texture 4	Texture 5	Texture 6
Ender-3 raw error	4.21±1.41	3.63±1.11	3.35±0.56	4.11±0.88	4.41±1.26	6.17±2.41
Ender-3 method error	1.96±0.36	2.79±1.56	1.87±0.27	1.90±0.30	2.06±0.43	2.18±0.56
Bambu raw error	2.52±0.45	2.39±0.19	2.99±0.69	2.19±0.34	2.54±0.40	2.02±0.30
Bambu method error	1.89±0.22	1.67±0.35	1.82±0.82	1.51±0.28	1.83±0.20	1.61±0.34
Resin raw error	2.78±1.46	4.10±1.27	3.01±1.47	2.27±0.30	2.42±0.12	2.96±0.63
Resin method error	2.01±0.25	2.04±0.95	2.05±0.51	2.23±0.57	2.21±0.17	1.84±0.90

**Table 3:** Average difference between repeat prints and the STL texture block. Metrics represent mean ICP distance (±standard deviation) in mm between scanned point clouds from each repeat and the STL block template for that texture, modified to a point cloud representation. Each scanned point cloud was aligned with the STL block via the iterative closest point (ICP) registration pipeline, following automated cropping and dynamic scaling of the scan. The ICP mean minimium distance is then taken as the difference metric. Samples with an ICP alignment threshold >1.5 mm were capped at 1.5 to avoid poor alignment, or a poor print, dominating the measure. We report the number of repeats, *x*, below the threshold as: n=x/5.

Printer Class	Texture 1	Texture 2	Texture 3	Texture 4	Texture 5	Texture 6
Ender-3	1.43±0.153, n=1/5	1.17±0.02, n=5/5	0.96±0.04, n=4/5	0.96±0.11, n=3/5	1.15±0.2, n=3/5	1.5±0, n=0/5
Bambu	0.59±0.5, n=4/5	0.90±0.02, n=5/5	0.69±0.45, n=5/5	0.99±0.28, n=5/5	0.35±0.05, n=5/5	0.84±0.37, n=4/5
Resin	0.96±0.48, n=4/5	0.84±0.38, n=5/5	0.61±0.5, n=5/5	0.89±0.06, n=3/5	0.42±0.02, n=5/5	0.66±0.03, n=4/5

**Table 4:** Data augmentation preliminary ablation study across different 3D printers, using 25 components with PCA. These data are for a Random Forest Classifier showing mean and standard deviation over 20 trials. The classifier was trained on batch 1 and tested on batch 2. This table contains data for the Ender-3 using PLA+, Bambu Lab using PLA-, and FormLabs Resin printer using grey V4.1.

Printer	Experiment	Accuracy (%)
**Ender-3**		
	No augmentation	54.15% ± 0.03
	Brightness	54.76% ± 0.03
	Noise	76.52% ± 0.03
	All augmentations	73.23% ± 0.02
**Bambu**		
	No augmentation	89.52% ± 0.02
	Brightness	89.5% ± 0.02
	Noise	91.65% ± 0.01
	All augmentations	90.24% ± 0.02
**Resin**		
	No augmentation	80.40% ± 0.03
	Brightness	79.99% ± 0.02
	Noise	94.98% ± 0.01
	All augmentations	94.50% ± 0.01

**Table 5:** Classification performance when trained on noise-only augmentation with PCA of 300 components. Training is done on 70% of one batch with testing on 30% of the same batch after augmentation. Train/Test accuracy are the final accuracy on these batches. The ‘Unseen print accuracy’ columns show the Classifier’s performance when evaluated on the larger set of print batches held out entirely from training, utilising the same printer and filament configuration, without augmentation. Results are gathered over 20 runs.

Classifier	Train Accuracy	Test Accuracy	Unseen Print Accuracy
RFC Ender-3	100.00% ± 0.00	98.66% ± 0.94	42.66% ± 2.25
ANN Ender-3	93.28% ± 0.06	91.79% ± 0.07	42.87% ± 0.03
RFC Bambu	100.00% ± 0.00	99.63% ± 0.75	73.75% ± 1.09
ANN Bambu	94.9%± 0.07	92.20% ± 0.10	57.46% ± 0.05
RFC Resin	100.00% ± 0.00	97.89% ± 1.99	80.27% ± 1.51
ANN Resin	92.28% ± 0.06	89.71% ± 0.07	56.35% ± 0.05

## Data Availability

Data for creating the benchmark can be accessed in the repository listed within Appendix A. Data for the TacTip movement can be found on Kaggle under the following links: https://www.kaggle.com/datasets/dextershepherd/repetative-3d-printed-dataset-bambu, accessed on 1 October 2026. https://www.kaggle.com/datasets/dextershepherd/repetative-dataset, accessed on 1 October 2026. https://www.kaggle.com/datasets/dextershepherd/repeatable-3d-dataset-resin, accessed on 1 October 2026.

## Supplementary Materials

The following supporting information can be downloaded at https://www.mdpi.com/article/10.3390/s26206590/s1, Table S1: Classification with PCA 300 performance when training on a single batch and evaluating on the training batch, and different printers. Results are reported as mean ± standard deviation. The results are averaged over 20 trials of the Random Forest Classifier; Table S2: Classification with raw images performance when training on a single batch and evaluating on the training batch, And different printers. Results are reported as mean ± standard deviation. The results are averaged over 20 trials of the Random Forest Classifier; Figure S1: PCA explained variance for the training batch of one printer, and the explained variance of the unseen test printers; Figure S2: Confusion matrix for the random forest classifier trained on the a: Bambu dataset, b: Resin dataset and c: Ender-3. All evaluated on the other printer batches; Figure S3: Results showing a heat map of the error between the STL point cloud (ground truth) and a scanned example. The scale shows red meaning more error and blue meaning less error. A and B show the resin print, C and D the Bambu, and E and F show the Ender-3.

## Appendix A. Repository

The code and experimental data are available at: https://github.com/shepai/3D-textures/, accessed on 1 October 2026. The naming conventions referred to in Section 2.5 correspond to:

- *3DD1*—Found in repository folder Generator/objects/
- *3DD2*—Found in repository file Generator/Generator.py
- *3DD3*—Found in repository readme section “Data & Experiments” part 1.

## Appendix B. Intel RealSense D405 Configuration

**Table A1:** Intel RealSense D405 configuration used in our experiments.

Property	Value
Device	Intel RealSense D405
Product Line	D400
Firmware Version	5.17.0.10
Depth Format	Z16
IR Format	Y8
Resolution	848×480
Frame Rate	15 FPS
Schema Version	1

**Table A2:** Intel RealSense D405 camera parameters.

Parameter	Value	Parameter	Value
Auto Exposure	True	Manual Exposure	33,000
Depth Gain	16	Auto White Balance	True
Depth Units	100	Z Units	100
Depth Clamp Min	0	Depth Clamp Max	65,536
Disparity Mode	0	Disparity Shift	0
Amplitude Factor	0	Lambda AD	800
Lambda Census	26	Left-Right Threshold	24
Median Threshold	500	Neighbor Threshold	7
Min Score Threshold A	1	Max Score Threshold B	2047
Second Peak Delta	325	Texture Count Threshold	0
Texture Difference Threshold	0	Use RSM	1
RAU Min E	1	RAU Min N	1
RAU Min S	1	RAU Min W	1
RAU Min NS Sum	3	RAU Min WE Sum	3
Region Shrink U	3	Region Shrink V	1
Region Color Threshold (R)	0.0499022	Region Color Threshold (G)	0.0499022
Region Color Threshold (B)	0.0499022	RSM Diff Threshold	4
RSM RAU SLO Diff Threshold	1	RSM Remove Threshold	0.375
Scanline P1	60	Scanline P2	342
Scanline P1 (1 Discon.)	105	Scanline P2 (1 Discon.)	190
Scanline P1 (2 Discon.)	70	Scanline P2 (2 Discon.)	130
Scanline Edge Tau (R)	72	Scanline Edge Tau (G)	72
Scanline Edge Tau (B)	72	Robbins–Monro Increment	10
Robbins–Monro Decrement	10	Ignore SAD	0
