# Supplementary material for: A 3D-Printable Dataset for Fair Testing and Comparisons of Tactile Sensors
Source: Sensors (Basel). 2026 Oct 17;26(20):6590. doi: 10.3390/s26206590 (PMC13708806; doi:10.3390/s26206590)
Supplement: Supplementary file 1 [file sensors-26-06590-s001.zip › sensors-4426528-supplementary.pdf]

Supplementary Material for A 3D-Printable Dataset for Fair Testing and Comparisons of Tactile Sensors

Dexter R. Shepherd, Nicolas Herzig, Phil Husbands,
Chris Johnson, Oluwaseyi Oladipupo Jesusanmi, William Kimbell,
Andrew Philippides

S1. Artifact Investigation

To test whether classifiers learn texture geometry independent of the fabrication process, we trained on all batches from one printer–material configuration and tested on the other two. Accuracy was below chance (16.7% for six classes) for Resin and Ender-3 unseen printers, The Ender achieved a higher accuracy than chance but still very low; Tables S1 and S2), despite near-perfect training accuracy. Thus classifiers trained on one configuration do not transfer to another using raw tactile images, and the within-configuration cross-batch results in the main paper should not be interpreted as evidence of cross-printer generalisation. Possible causes include configuration-specific surface artifacts and dif-ferences in material appearance affecting sensor images; the present design cannot separate these.

**Training printer**	**Training batch**	**Other printers**
Ender	$100.00 \pm 0.00\%$	Resin: $16.65 \pm 1.33\%$<br>Bambu: $36.72 \pm 1.31\%$
Bambu	$100.00 \pm 0.00\%$	Resin: $14.46 \pm 1.59\%$<br>Ender: $24.21 \pm 1.34\%$
Resin	$100.00 \pm 0.00\%$	Ender: $13.03 \pm 3.68\%$<br>Bambu: $22.90 \pm 2.36\%$

Table S1: Classification with PCA 300 performance when training on a single batch and evaluating on the training batch, and different printers. Results are reported as mean ± standard deviation. The results are averaged over 20 trials of the Random Forest Classifier

**Training printer**	**Training batch**	**Other printers**
Ender	$100.00 \pm 0.00\%$	Resin: $15.65 \pm 1.82\%$<br>Bambu: $33.14 \pm 1.31\%$
Bambu	$100.00 \pm 0.00\%$	Resin: $14.87 \pm 1.39\%$<br>Ender: $24.91 \pm 1.46\%$
Resin	$100.00 \pm 0.00\%$	Ender: $8.60 \pm 4.61\%$<br>Bambu: $23.03 \pm 3.04\%$

Table S2: Classification with raw images performance when training on a single batch and evaluating on the training batch, And different printers. Results are reported as mean ± standard deviation. The results are averaged over 20 trials of the Random Forest Classifier

To understand why classifiers did not transfer across configurations, we compared how much of the variance in held-out tactile images was captured by a PCA basis fitted on the training configuration. For held-out batches from the same configuration, the basis captured approximately 90% of the variance (a gap of 1̃0% relative to the training set. For prints from a different printer-material configuration, the captured variance was 50-60% and did not approach the training level even with 2,000 components, whereas PCA fitted to each held-out configuration separately captured ¿95% with

$\tilde{3}00$ components (Figure S1). Tactile images from different configurations are therefore similarly low-dimensional but occupy different subspaces. This is consistent with the near-chance cross-configuration accuracy (Table S1), where predictions collapsed onto a single class, as shown in the confusion matrices in Figure S2.

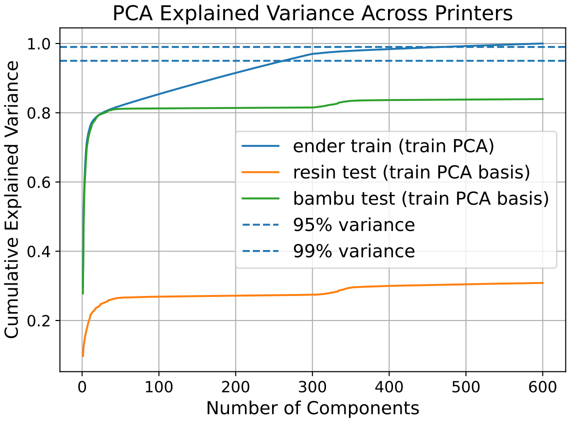

(a) Ender-3

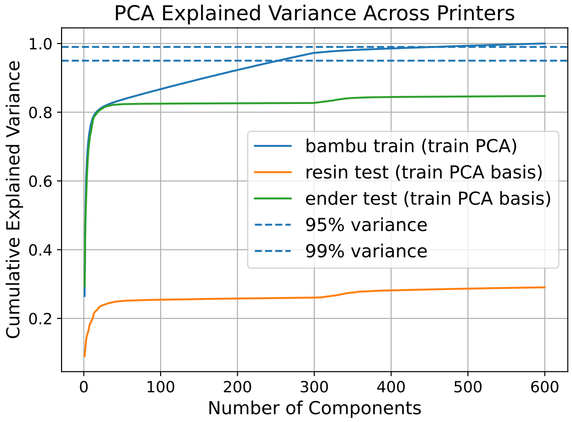

(b) Bambu

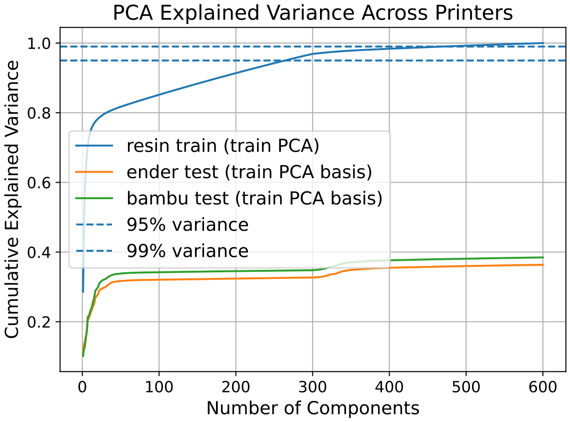

(c) Resin

Figure S1: PCA explained variance for the training batch of one printer, and the explained variance of the unseen test printers.

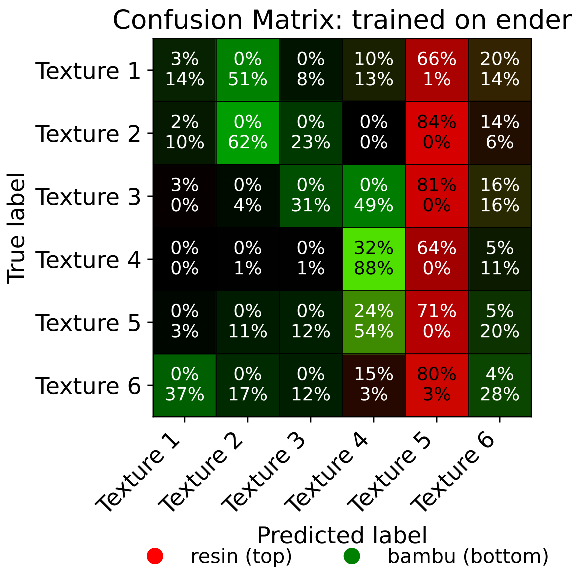

(a) Ender-3

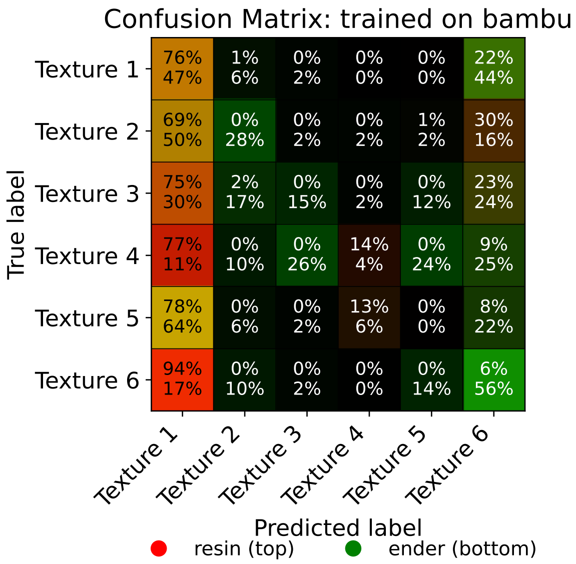

(b) Bambu

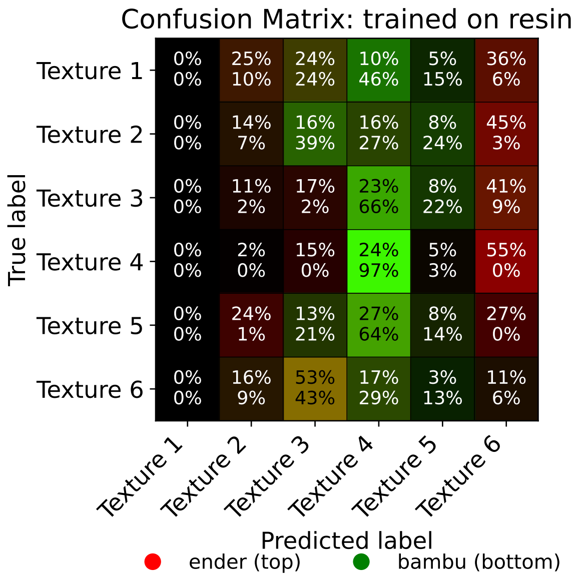

(c) Resin

Figure S2: Confusion matrix for the random forest classifier trained on the a: Bambu dataset, b: Resin dataset and c: Ender-3. All evaluated on the other printer batches.

Classifiers trained on the Ender-3/PLA+ configuration, which had the highest between-batch variability, transferred marginally better to other configurations than those trained on the more repeatable Bambu or Resin prints, although all remained close to chance (Table S1). One possible explanation is that greater variability in the training prints exposes the classifier to a wider range of tactile responses per texture, acting as an implicit form of augmentation. We have not tested this explanation, and the differences are small relative to run-to-run variation.

To determine whether it is a PCA transfer issue, we trained the classifiers on the raw images without PCA.

S2. STL comparison

To further highlight the error across printers and batches from the STL truth, Figure S3 shows that the Ender-3 struggles the most against noise.

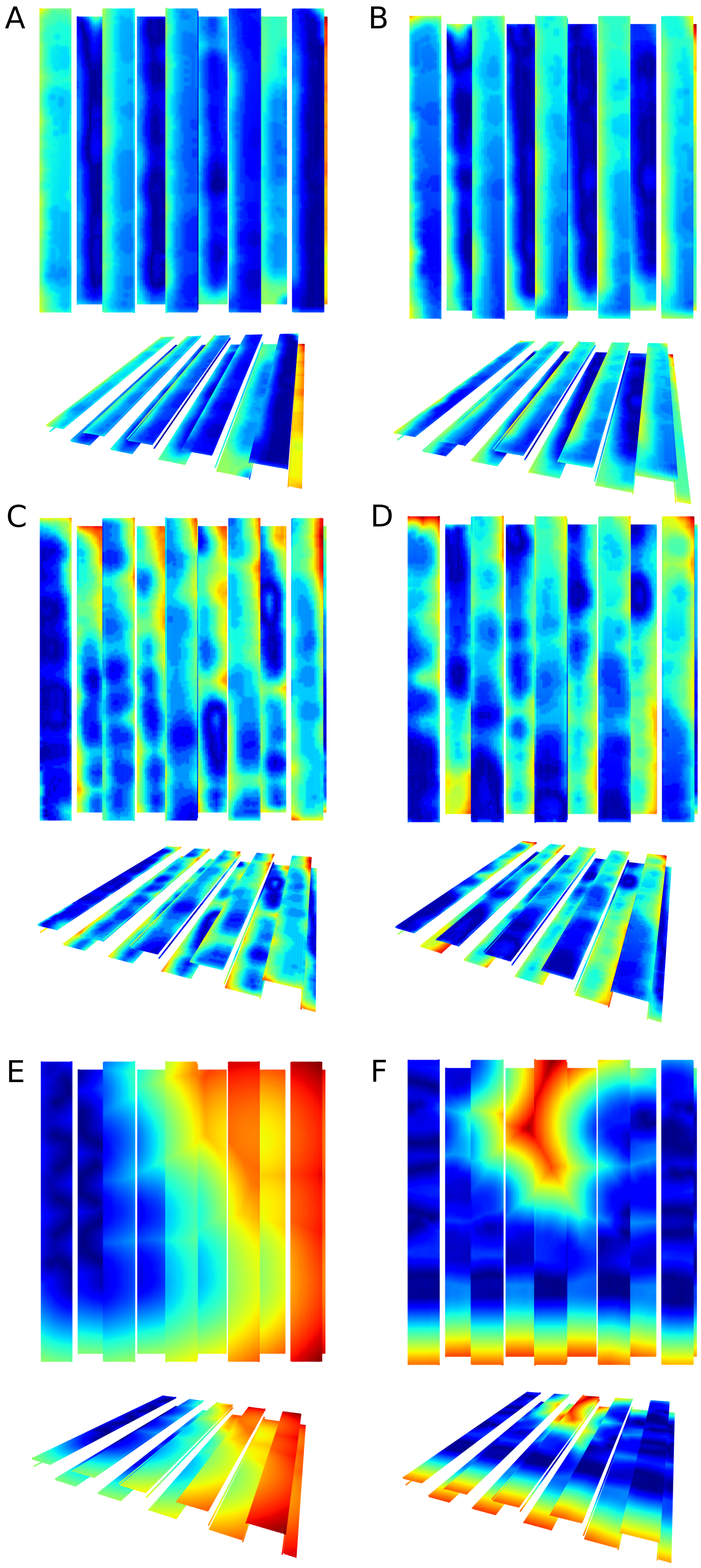

Figure S3: Results showing a heat map of the error between the STL point cloud (ground truth) and a canned example. The scale shows red meaning more error and blue meaning less error. A and B show the resin print, C and D the Bambu, and E and F show the Ender-3.
